# The Influence of Diet and Sex on the Gut Microbiota of Lean and Obese JCR:LA-*cp* Rats

**DOI:** 10.3390/microorganisms9051037

**Published:** 2021-05-12

**Authors:** Craig Resch, Mihir Parikh, J. Alejandro Austria, Spencer D. Proctor, Thomas Netticadan, Heather Blewett, Grant N. Pierce

**Affiliations:** 1 Department of Physiology and Pathophysiology, Faculty of Health Sciences, University of Manitoba, Winnipeg, MB R2H 2A6, Canada; cresch@sbrc.ca (C.R.); mihir.parikh@mssm.edu (M.P.); aaustria@sbrc.ca (J.A.A.); tnetticadan@sbrc.ca (T.N.); 2 The Institute of Cardiovascular Sciences, Winnipeg, MB R2H 2A6, Canada; 3 Canadian Centre for Agri-food Research in Health and Medicine (CCARM), St. Boniface Hospital Albrechtsen Research Centre, 351 Taché Avenue, Winnipeg, MB R2H 2A6, Canada; hblewett@sbrc.ca; 4 Metabolic and Cardiovascular Diseases Laboratory, Division of Human Nutrition, University of Alberta, Edmonton, AB T6G 2E1, Canada; proctor@ualberta.ca; 5 Agriculture and Agri-Food Canada, St. Boniface Hospital Albrechtsen Research Centre, 351 Taché Avenue, Winnipeg, MB R2H 2A6, Canada; 6 Department of Food and Human Nutritional Sciences, Faculty of Agricultural and Food Sciences, University of Manitoba, Winnipeg, MB R2H 2A6, Canada

**Keywords:** gut microbiota, short-chain fatty acids, obesity

## Abstract

There is an increased interest in the gut microbiota as it relates to health and obesity. The impact of diet and sex on the gut microbiota in conjunction with obesity also demands extensive systemic investigation. Thus, the influence of sex, diet, and flaxseed supplementation on the gut microbiota was examined in the JCR:LA-*cp* rat model of genetic obesity. Male and female obese rats were randomized into four groups (*n* = 8) to receive, for 12 weeks, either (a) control diet (Con), (b) control diet supplemented with 10% ground flaxseed (CFlax), (c) a high-fat, high sucrose (HFHS) diet, or (d) HFHS supplemented with 10% ground flaxseed (HFlax). Male and female JCR:LA-*cp* lean rats served as genetic controls and received similar dietary interventions. Illumine MiSeq sequencing revealed a richer microbiota in rats fed control diets rather than HFHS diets. Obese female rats had lower alpha-diversity than lean female; however, both sexes of obese and lean JCR rats differed significantly in β-diversity, as their gut microbiota was composed of different abundances of bacterial types. The feeding of an HFHS diet affected the diversity by increasing the phylum Bacteroidetes and reducing bacterial species from phylum Firmicutes. Fecal short-chain fatty acids such as acetate, propionate, and butyrate-producing bacterial species were correspondingly impacted by the HFHS diet. Flax supplementation improved the gut microbiota by decreasing the abundance of Blautia and *Eubacterium dolichum*. Collectively, our data show that an HFHS diet results in gut microbiota dysbiosis in a sex-dependent manner. Flaxseed supplementation to the diet had a significant impact on gut microbiota diversity under both flax control and HFHS dietary conditions.

## 1. Introduction

Studies understanding the association between human health and the gut microbiota have received significant interest in the last decade [1]. The link between gut microbiota dysbiosis and disease development remains unclear, and it is still unknown whether adverse metabolic changes precede or follow the alterations in the composition of gut microbiota [2]. The gut microbiota plays a crucial role in various functions of the digestive tract such as: (a) maintaining the intestinal epithelial integrity, and thus protecting against pathogenic bacteria [3], (b) metabolizing dietary fiber and helping in the absorption of short-chain fatty acids (SCFAs) from indigestible fiber by bacterial fermentation [4], and (c) regulating intestinal transit, hence impacting the absorbable energy from the diet [5]. These essential roles highlight the importance of gut microbiota in metabolism and body weight. Obesity is a major public health concern and is increasingly being tied to the changes in gut microbiota composition [6]. Several studies have reported altered gut microbiota composition and reduced microbial diversity in obese individuals compared to normal weight adults [7,8,9]. Furthermore, compared to lean counterparts, adults with obesity also have specific bacterial groups enriched or decreased [10]. Studies have indicated that obesity associated metabolic abnormalities may also be attributed to the dysbiosis of gut microbiota [11]. Targeting the gut microbiota using diets [12], probiotics [13], prebiotics [14], and drugs [15] have improved metabolic conditions.

Dietary changes also have the capacity to rapidly modify gut microbiota both in animals [16] and humans [17]. High-fat diet (HFD) consumption contributes to obesity, gut microbiota dysbiosis, and metabolic syndrome [18]. In multiple disease models, HFD can also alter the gut microbial environment [19]. HFD has been reported to increase the levels of phyla Firmicutes and Proteobacteria and decrease beneficial species of *Bifidobacterium* spp. and *Lactobacillus gasseri* [20]. The body fat percent growth in a high-fat high-sucrose (HFHS) diet fed to mice was negatively associated with an excess of Akkermansia (phylum Verrucomicrobia) and was positively related to Lactococcus (phylum Firmicutes) and Allobaculum (phylum Bacteroidetes) [21]. Furthermore, a HFHS diet was capable of inducing gut microbial dysbiosis in more than 200 strains of mice with genetic variations [22]. This suggests that dietary perturbations can produce changes in the gut microbiota despite variations in the host genome. Diet-responsive bacterial groups in gut microbiota respond to dietary shifts within an average of 3.5 days to reach a new steady state [22].

Flaxseed, *Linum usitatissimum*, is a rich dietary plant source of the essential omega-3 polyunsaturated fatty acid alpha-linolenic acid (ALA), the lignan secoisolariciresinol diglucoside (SDG), and dietary fiber [23]. Proteobacteria and Porphyromonadaceae respond to flax oil [24], whereas Bifidobacterium and Faecalibacterium are sensitive to flax fiber [25,26]. SDG is particularly susceptible to the gut microbiota as it is metabolized by intestinal bacteria including *Ruminococcus bromii*, *Ruminococcus lactaris, Lactobacillus casei, and Lactobacillus acidophilus* to become bioavailable in the form of enterodiol and enterolactone [27,28]. Flaxseed supplementation in the diet of rodents can alter the profile and diversity of *Enterobacteriaceae* [29], *Prevotella* spp. [30], *Akkermansia muciniphila* [30], and *Bifidobacterium* [31]. However, our understanding of the effect of dietary flaxseed and HFHS on the gut microbiota in an obese environment with sex as another independent variable is unknown. We, therefore, investigated the effect of a HFHS diet and flax feeding on a JCR:LA-*cp* rat model of genetic obesity in both male and female animals. The JCR:LA-*cp* rat has an autosomal recessive leptin receptor mutation [32]. Thus, JCR:LA-*cp* rats manifest an obese phenotype, along with metabolic syndrome-related abnormalities such as dyslipidemia, hepatic injury, atherosclerosis, and cardiac dysfunction [32,33,34,35].

In this study we investigated: (a) the differences in the composition of gut microbiota between lean and obese genotypes of male and female JCR:LA-*cp* rats fed control, HFHS and/or flax supplemented diets, (b) the effect of a HFHS and/or flax supplemented diets on the gut SCFA profile of male and female JCR:LA-*cp* rat strain of both genotypes.

## 2. Materials and Methods

### 2.1. Animal Model and Experimental Protocol

This study protocol (18-022) was approved by the University of Manitoba Office of Research Ethics & Compliance and was done in accordance with the guidelines by the Canadian Council for Animal Care. Obese (*cp/cp*) and lean (*cp/?*) JCR:LA-*cp* rats were raised from the colony established at the University of Alberta [33,34]. Male and female animals of both genotypes were housed in a temperature and humidity-controlled room with a 12 h light/dark cycle. At 12 weeks of age, obese JCR:LA-*cp* rats of both sexes (each *n* = 32) were randomized to receive either (a) regular chow diet (Con), (b) 10% ground flaxseed supplemented regular chow diet (CFlax), (c) HFHS, or (d) 10% ground flaxseed supplemented HFHS diet (HFlax), for 12 weeks. Age-matched male and female lean (*cp/?*) JCR rats served as control animals and were given similar diets for 12 weeks.

The regular chow was a Prolab^®^ RMH 3000 regular rodent chow, and the HFHS included AIN-93G chow with 35% fat (lard) and 36% carbohydrate (mostly sucrose) (TestDiet, Richmond, IN, USA). The ground flaxseed was BakePur milled flaxseed obtained from Pizzey Ingredients, Russell, Manitoba, Canada. Throughout the study duration, animals had free access to water and food.

### 2.2. Biological Sample Collection

After 12 weeks of feeding, 24-week old animals were fasted overnight (16 h) and then anesthetized with isoflurane (5%) the following morning. The depth of anesthesia was assessed by a pedal withdrawal reflex. After anesthesia, the blood sample was collected from the inferior vena cava by opening the thoracic cavity and the heart was immediately excised. One or two fecal pellets were removed from the distal colon of each animal. Samples were snap frozen in liquid nitrogen and stored at −80 °C until further analysis.

### 2.3. Illumina MiSeq Sequencing of 16S rRNA Gene V4 Region of Gut Microbiota

All fecal samples were analyzed in the Microbiome Insights Laboratory (Vancouver, BC, Canada). The fecal pellets were placed into a MoBio PowerMag Soil DNA Isolation Bead Plate. Total genomic DNA of the gut microbiota was extracted from fecal samples by following MoBio’s instructions on a KingFisher robot. Bacterial 16S rRNA genes were PCR-amplified with dual-barcoded primers targeting the V4 region (515F 5′-GTGCCAGCMGCCGCGGTAA-3′, and 806R 5′-GGACTACHVGGGTWTCTAAT-3′), as previously described [36]. Amplicons were sequenced with an Illumina MiSeq using the 300-bp paired-end kit (v.3). Sequences were denoised, taxonomically classified using Greengenes (v. 13_8) as the reference database, and clustered into 97%-similarity operational taxonomic units (OTUs) using the mothur software package (v. 1.39.5) [37], following the recommended procedure (https://www.mothur.org/wiki/MiSeq_SOP; accessed on November 2017). The analytical flowchart is illustrated in Supplementary Figure S1.

### 2.4. Quality Control

The possibility of contamination was investigated by co-sequencing DNA amplified from feces samples and from 7 each of template-free controls and extraction kit reagents processed in the same manner as the specimens. Operational taxonomic units were considered putative contaminants (and were removed) if their mean abundance in controls reached or exceeded 25% of their mean abundance in specimens.

### 2.5. Short Chain Fatty Acid Measurement

SCFAs acids were extracted from the feces samples and derivatized as previously described [38]. Extracted SCFA supernatants were stored in 2-mL GC vials, with glass inserts. SCFA were detected using gas chromatography (Thermo Trace 1310, Thermo Fisher Scientific, Waltham, MA, USA) using Thermo TG-WAXMS A GC Column, 30 m, 0.32 mm, 0.25 µm coupled to a flame ionization detector (Thermo). The following settings were used for detection: Flame ionization detector temperature was kept at 240 °C, hydrogen at 35.0 mL/min, air at 350.0 mL/min, makeup gas (Nitrogen) at 40.0 mL/min Inlet, and carrier pressure at 225 kPa. Column flow was set at 6.00 mL/min with purge flow of 5.00 mL/min and split flow of 12.0 mL/min at temperature 200 °C.

### 2.6. Sequence Metrics and Taxonomic Composition

The microbiota was analyzed by sequencing the 16Sv4 rRNA amplicons generated from the fecal pellets of 24-week-old JCR:LA-*cp* rats. Miseq generated high quality filtered files were clustered into 122,196 operational taxonomic units (OTUs) at a similarity cutoff of 97%. An average of 44,497 quality-filtered reads were generated per sample (Table S1 and Figure S2). High quality reads were classified using Greengenes v. 13_8 as the reference database. OTUs were aggregated into each taxonomic rank.

### 2.7. Statistical Analysis

Alpha diversity was estimated with the Shannon index on OTU abundance tables after filtering out contaminants and rarefaction using a minimum total count of (19,443). The significance of diversity differences was tested with three-way ANOVA (Y = α + β1? Sex + β2? Diet + β3? Genotype + β12? Sex × Diet + β13? Sex × Genotype + β23? Diet × Genotype + β123? Sex × Diet × Genotype). Tukey post-hoc test determined significant pairwise differences. To estimate β-diversity across samples, we excluded OTUs occurring with a count of less than 3 in at least 10% of the samples and then computed Bray-Curtis indices. We visualized β-diversity, emphasizing differences across samples, using Principal Coordinate Analysis (PCoA) ordination. Variation in community structure was assessed with permutational multivariate analyses of variance (PERMANOVA) with treatment group as the main fixed factor and using 9999 permutations for significance testing. All analyses were conducted in the R environment. Pairwise contrasts were tabulated and the FDR method used to correct *p*-values for multiple comparisons. *DESeq2* package was used to identify differentially abundant taxa among Sex, Diet and Genotype variables. The following linear model was used for the test: ~Sex x Diet x Gentoype, and the reduced terms of the *likelihood ratio test* are: ~1.

## 3. Results

### 3.1. Diet, Sex, and Genotype Alters the Composition and Diversity of Gut Microbial Ecology

**Figure 1:**
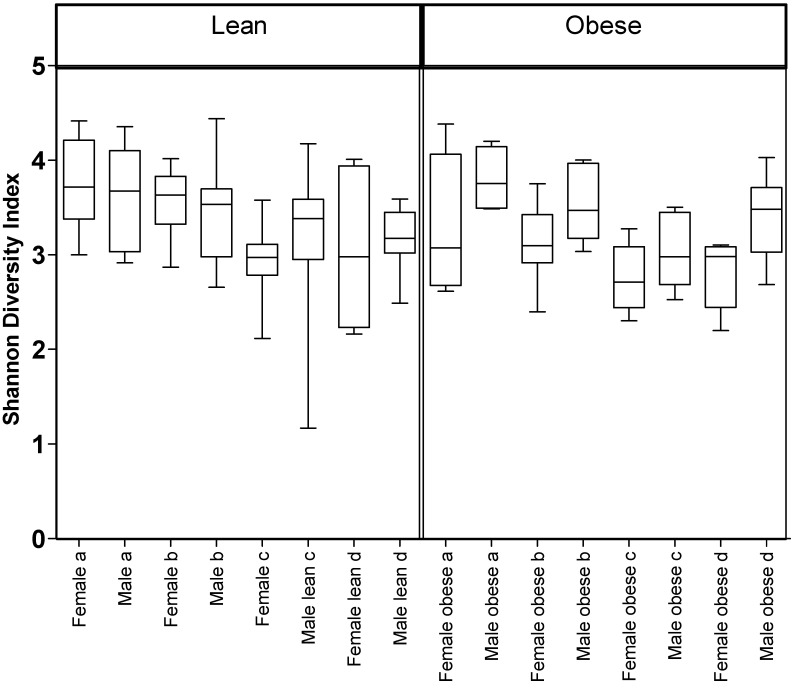
Alpha diversity of gut microbiota. Shannon diversity plot from fecal pellets of lean and obese, male or female JCR:LA-*cp* rats fed (a) control diet (Con), (b) control + flax (CFlax), (c) high fat, high sucrose (HFHS) or (d) high fat, high sucrose + flax (HFlax). Con and HFHS (*p* < 0.001), Con and HFlax (*p* < 0.001), CFlax and HFHS (*p* < 0.003), male and female (*p* < 0.016), lean females and obese females (*p* < 0.015). *n* = 8.

The alpha-diversity (Shannon index) was calculated for each sample (Figure 1). This diversity is a measure of richness (number of OTUs) and evenness (even distribution of OTUs) in a sample. A three-way ANOVA revealed differences in the Shannon diversity index with significant main effect of diet (*p* < 0.001). Consumption of a HFHS diet resulted in decreased microbial diversity. Animals on the HFHS diet had significantly lower Shannon index compared to animals on the Con (*p* < 0.0001) or CFlax diet (*p* < 0.001). No significant difference in the diversity between CFlax and HFlax diet groups (*p* < 0.087) was noted. There was a significant difference in Shannon diversity between sexes (*p* < 0.016). Obese female JCR:LA-*cp* rats had lower values for Shannon diversity index compared to lean females (*p* < 0.015), but there was no significant difference in the diversity between obese and lean males (*p* < 0.495).

**Figure 2:**
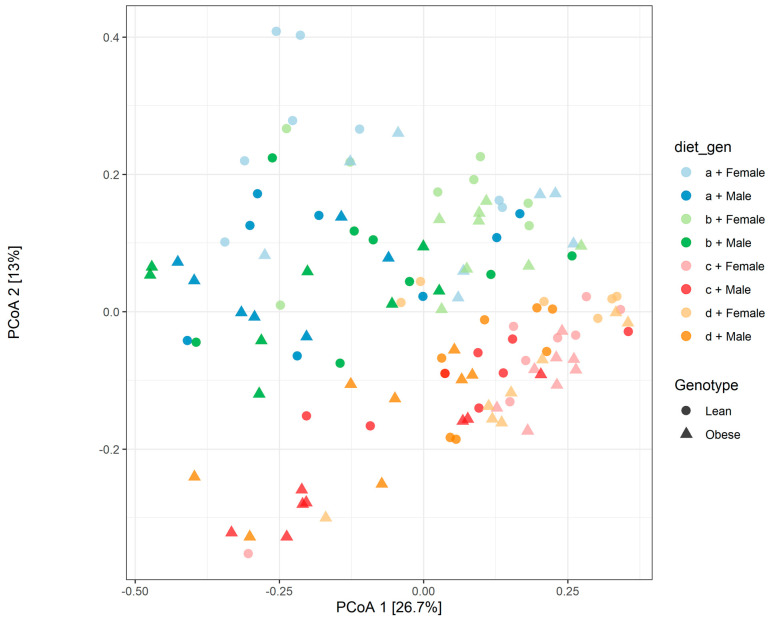
β-diversity of gut microbiota. PCoA ordination plot for β-diversity of microbiota among lean and obese, male or female JCR:LA-*cp* rats fed (a) control diet, (b) control + flax, (c) high fat, high sucrose or (d) high fat, high sucrose + flax. Genotype (R2 = 0.035, *p* < 0.0001), diet (R2 = 0.1624, *p* < 0.0001), sex (R2 = 0.0705, *p* < 0.0001), sex and diet (R2 = 0.0314, *p* < 0.0077), sex and genotype (R2 = 0.0336, *p* < 0.0001), diet and genotype (R2 = 0.0305, *p* < 0.0001). *n* = 8.

We summarized OTU abundances into Bray–Curtis dissimilarities and performed a principal component analysis (PCoA) ordination. The PCoA ordination plot assesses whether distinct clusters of the relative abundance of gut microbiota are produced as an impact of diet, genotype, or sex. This generates a graphical representation of microbiota composition dissimilarity among samples (β-diversity). A clear cluster of gut microbiota from obese male and female animals on the HFHS and HFlax diet was observed separated from the Con and the CFlax diet (Figure 2). Accordingly, a PERMANOVA determined a significant difference in β-diversity among genotype, diet, and sex. Obese and lean animals had an adonis R2 = 0.035, *p* < 0.0001, and there is a significant difference among diet with an adonis R2 = 0.1624, *p* < 0.0001. The PCoA plot for β-diversity also shows a separation among male and female (Figure 2) with an adonis R2 = 0.0705, *p* < 0.0001. There were also significant differences in β-diversity among sex and diet (R2 = 0.0314, *p* < 0.0077), sex and genotype (R2 = 0.0336, *p* < 0.0001) and diet and genotype (R2 = 0.0305, *p* < 0.0001).

**Figure 3:**
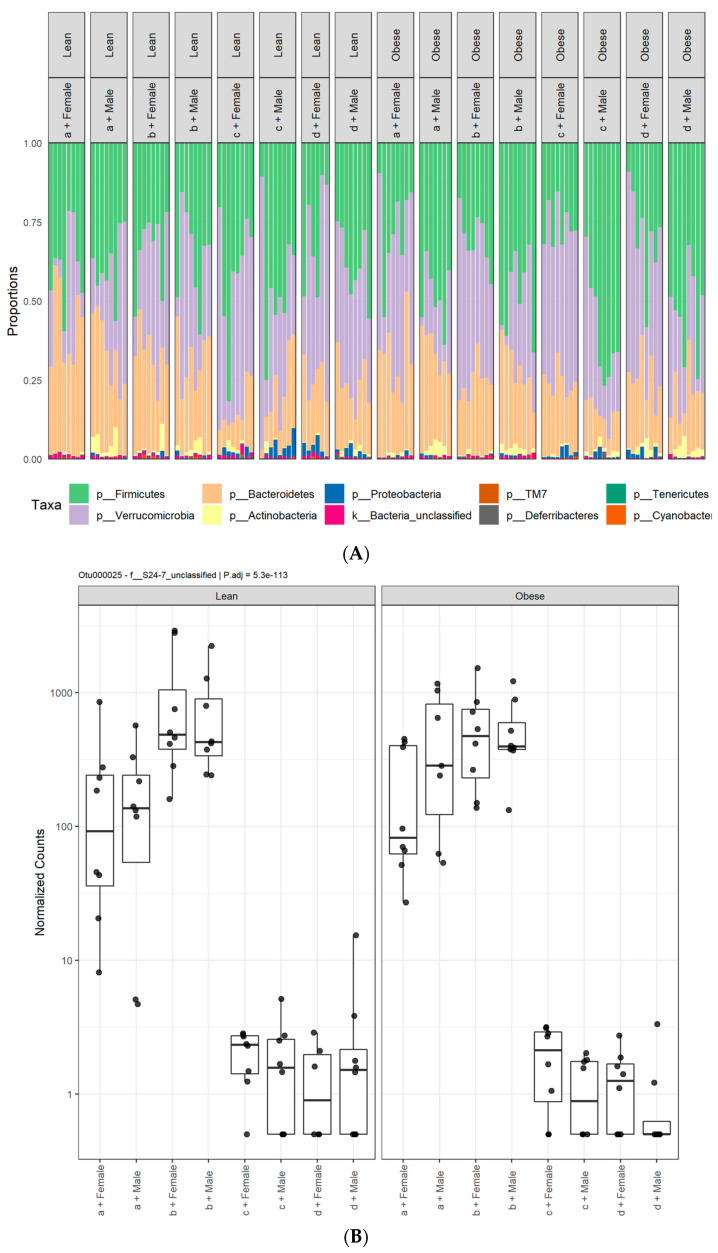

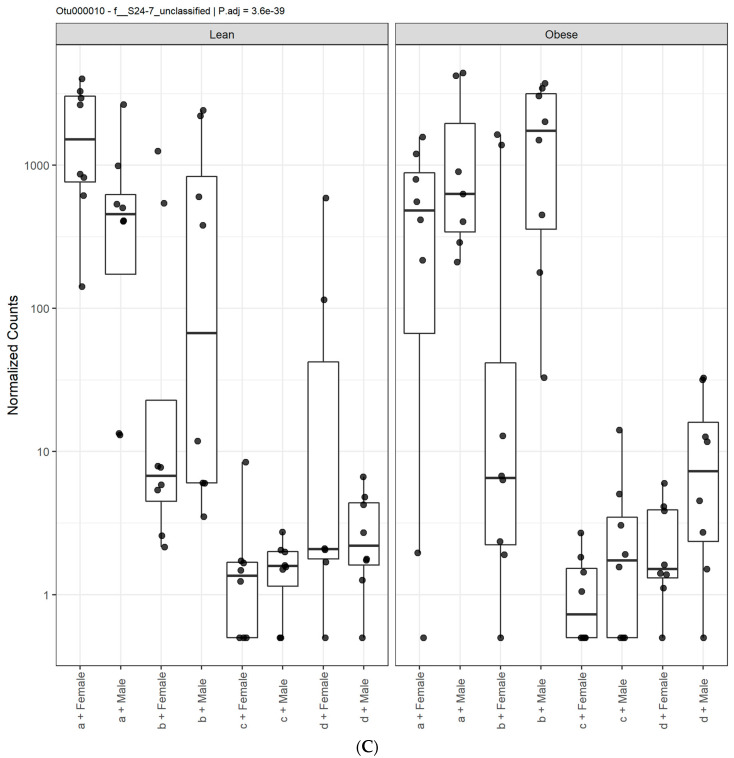
Variation in gut microbiota diversity at the phylum level. (**A**), Taxonomical analysis of variations in gut microbiota; (**B**) OTU000025; (**C**) OTU000010 are species from the phylum Bacteroidetes that are differentially abundant among lean and obese, male or female JCR:LA-*cp* rats fed (a) control diet, (b) control + flax, (c) high fat, high sucrose or (d) high fat, high sucrose + flax. *n* = 8.

**Figure 4:**
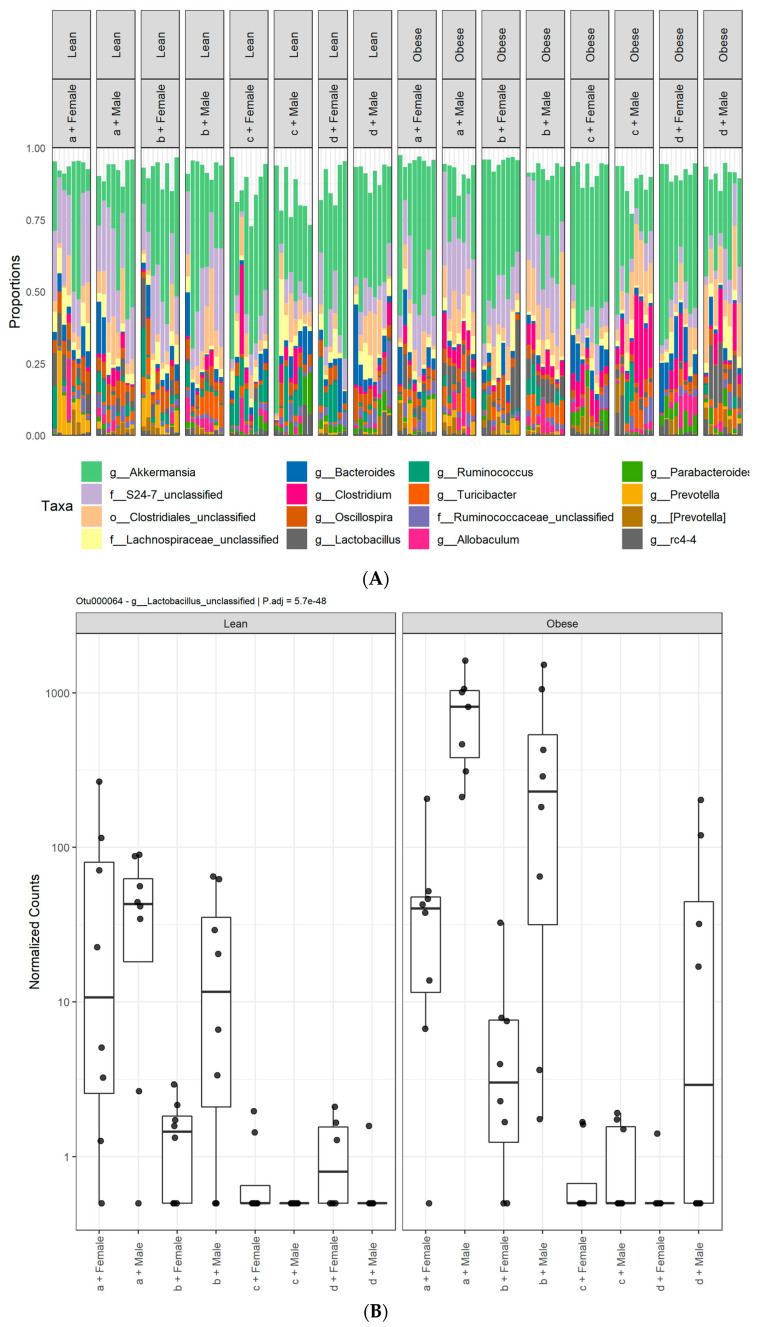

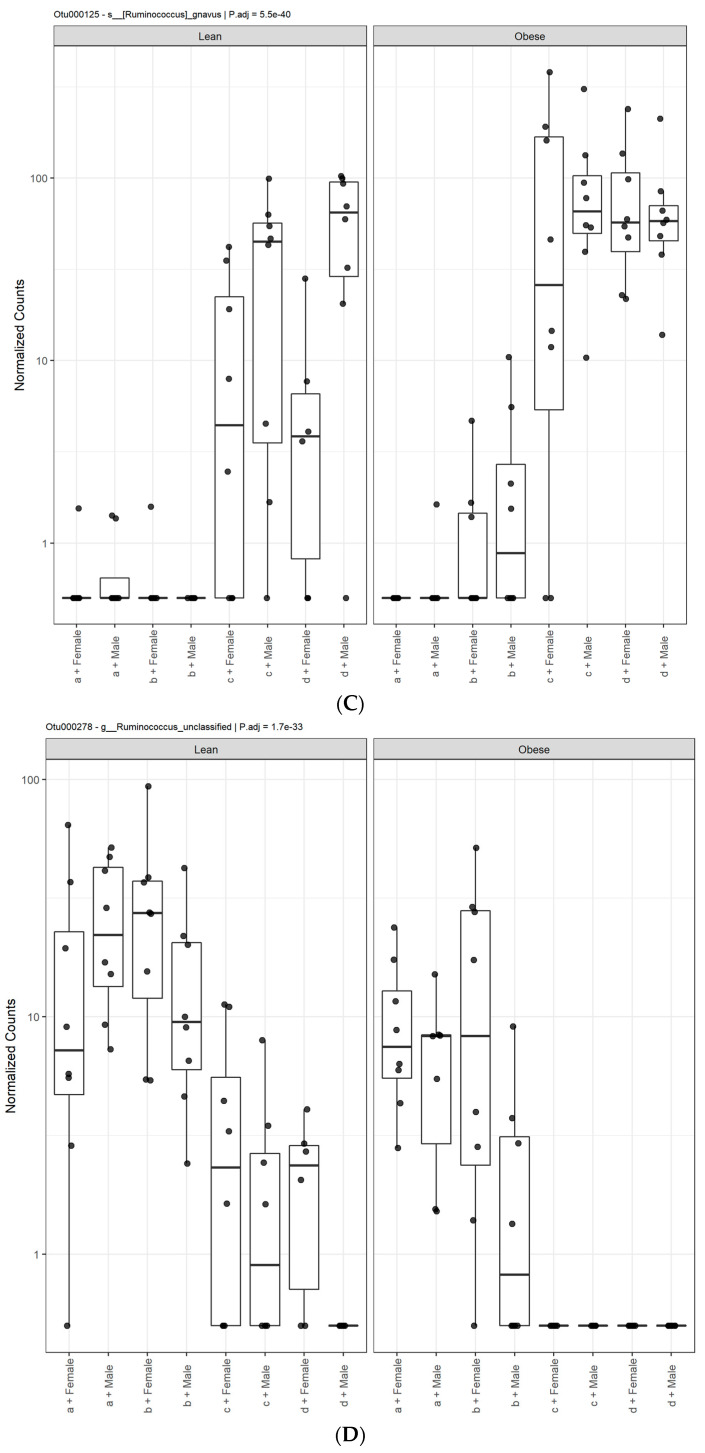

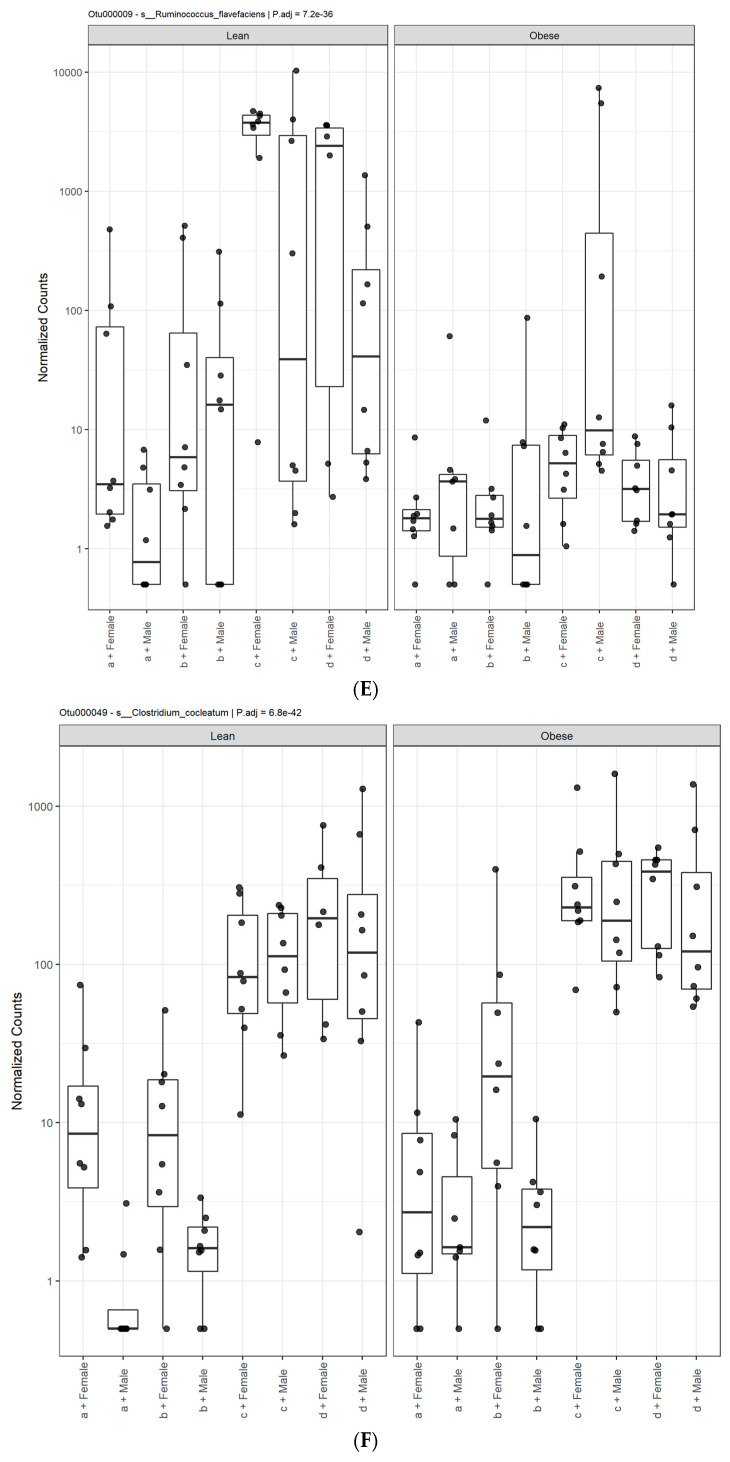

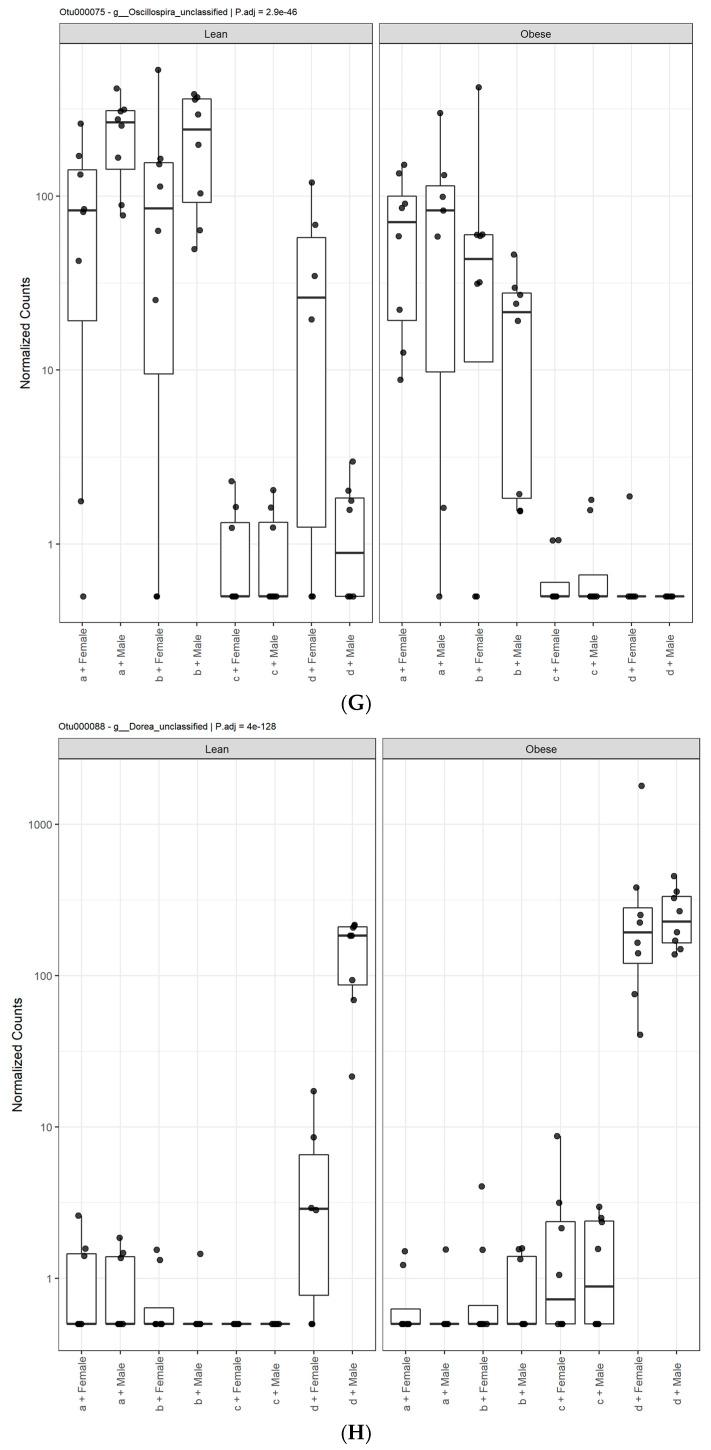

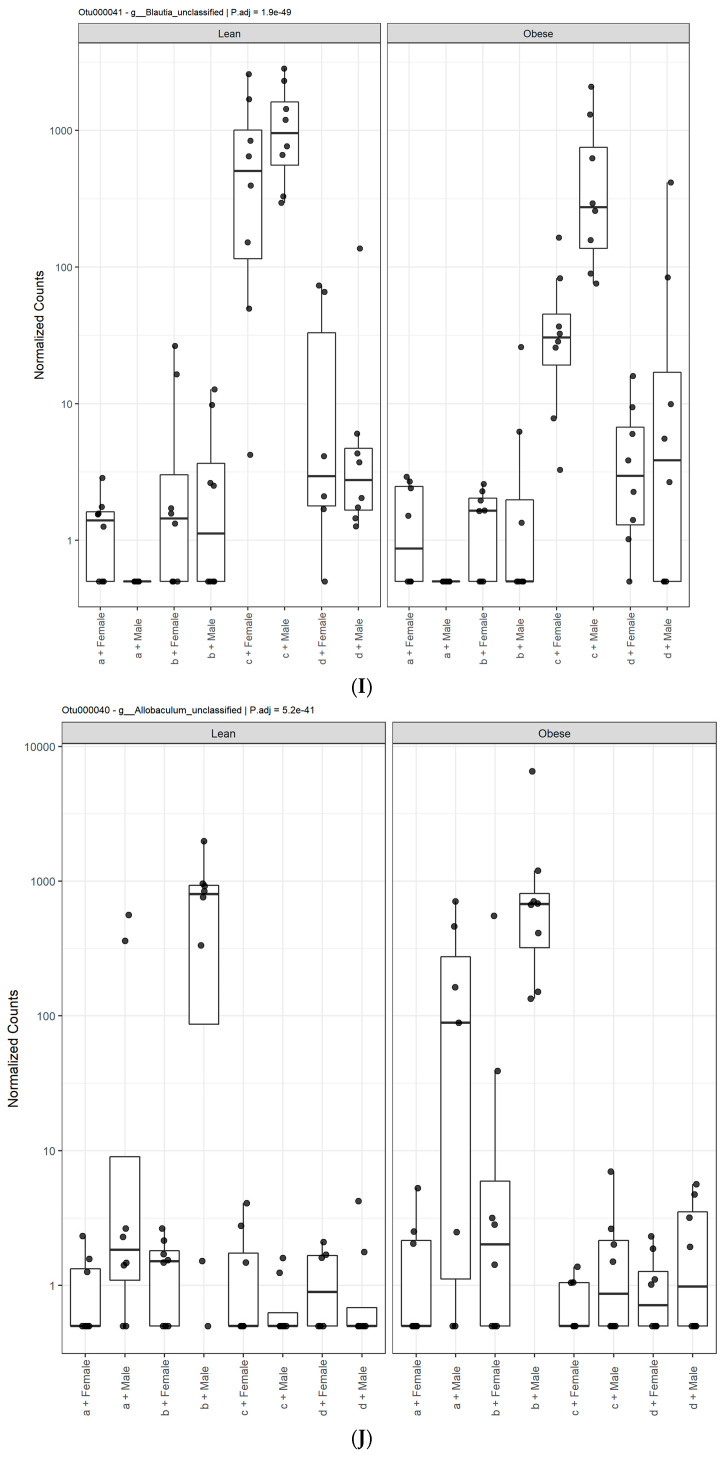

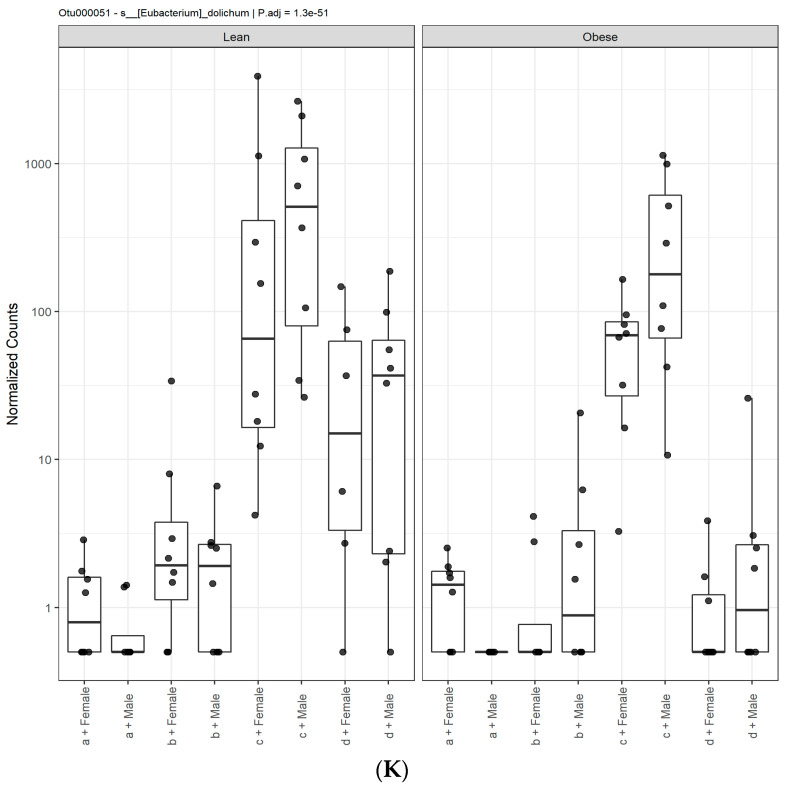
Relative abundance of gut microbiota at the genera level. (**A**), Taxonomical analysis of select microbial genera. Relative abundance of Firmicutes (**B**), *Lactobacillus* sp.; (**C**), *Ruminococcus gnavus*; (**D**), *Ruminococcus* unclassified; (**E**), *Ruminococcus flavefaciens*; (**F**), *Clostridium cocleatum*; (**G**), *Oscillospira* sp.; (**H**), *Dorea* sp.; (**I**) *Blautia* sp.; (**J**) *Allobaculum* sp.; (**K**) *Eubacterium dolichum*; that are differentially abundant among lean and obese, male or female JCR:LA-*cp* rats fed (a) control diet, (b) control + flax, (c) high fat, high sucrose or (d) high fat, high sucrose + flax. *n* = 8.

The four most abundant phyla observed in all the fecal samples were Actinobacteria, Bacteroidetes, Firmicutes, and Verrucomicrobia (Figures S3–S7). Taxonomic based analysis of the relative abundance of the gut microbiota revealed variances among the treatments at the phylum level (Figure 3A). A *DESeq2* package was used to identify differentially abundant taxa among diet, sex and genotype variables. The phylum Bacteroidetes had two different unclassified species from the family Muribaculaceae (previously known as S24-7) (OTU000025, P.adj = 5.3e-113 and OTU000010, P.adj = 3.6e-39) that were significantly lower in rats fed HFHS and HFlax diets compared to Con and CFlax diets (Figure 3B,C). The relative abundance of gut microbiota also varied at the genus level (Figure 4A). There were 13 bacterial genera from the phylum Firmicutes that were significantly differentially abundant. The abundance of many of these bacteria was affected by the HFHS diet compared to the Con diet. A *Lactobacillus* species (P.adj = 5.7e-48) had a very low abundance in HFHS and HFlax fed rats compared to the Con and CFlax fed rats (Figure 4B). Three bacterial species from the genus *Ruminococcus* were differentially abundant. *Ruminococcus gnavus* (P.adj = 5.5e-40) and *Ruminococcus* unclassified (P.adj = 1.7e-33) had a lower abundance in the HFHS group. Conversely, *Ruminococcus flavefaciens* (P.adj = 7.2e-36) was slightly elevated in the HFHS group (Figure 4C–E). A higher abundance of *Clostridium cocleatum* (P.adj = 6.8e-42) was observed in rats fed the HFHS diet compared to the Con diet (Figure 4F) while a lower abundance of an *Oscillospira* species (P.adj = 2.9e-46) was observed in the HFHS (Figure 4G). A higher abundance of an unclassified species from the family *Lachnospiraceae* was observed in rats fed the HFHS diet (Figure S10).

Flaxseed supplementation in the HFlax diet group also differentially affected the abundance of bacterial species. A *Dorea* species (P.adj = 4e-128) was elevated only in rats fed HFlax, but not in HFHS fed rats (Figure 4H). A *Blautia* species (P.adj = 1.9e-49) was significantly elevated in the HFHS diet compared to the Con diet. Flax supplementation significantly lowered a *Blautia* species compared to the HFHS group (Figure 4I). An *Allobaculum* species (P.adj = 5.2e-41) was elevated in lean and obese males fed the CFlax diet (Figure 4J). *Eubacterium dolichum* (P.adj = 1.3e-51) was elevated in the HFHS diet compared to the Con group. This was significantly reduced in obese rats fed the HFlax diet (Figure 4K). *Subdoligranulum variabile* (P.adj = 6.2e-36) and a *SMB53* species (P.adj = 6.2e-48) were significantly high in the obese animals on the HFHS diet and flax supplementation in the HFlax diet reduced the abundance of these bacteria (Figures S8 and S9).

### 3.2. Diet, Sex, and Genotype Impacts the Gut SCFA Composition

**Figure 5:**
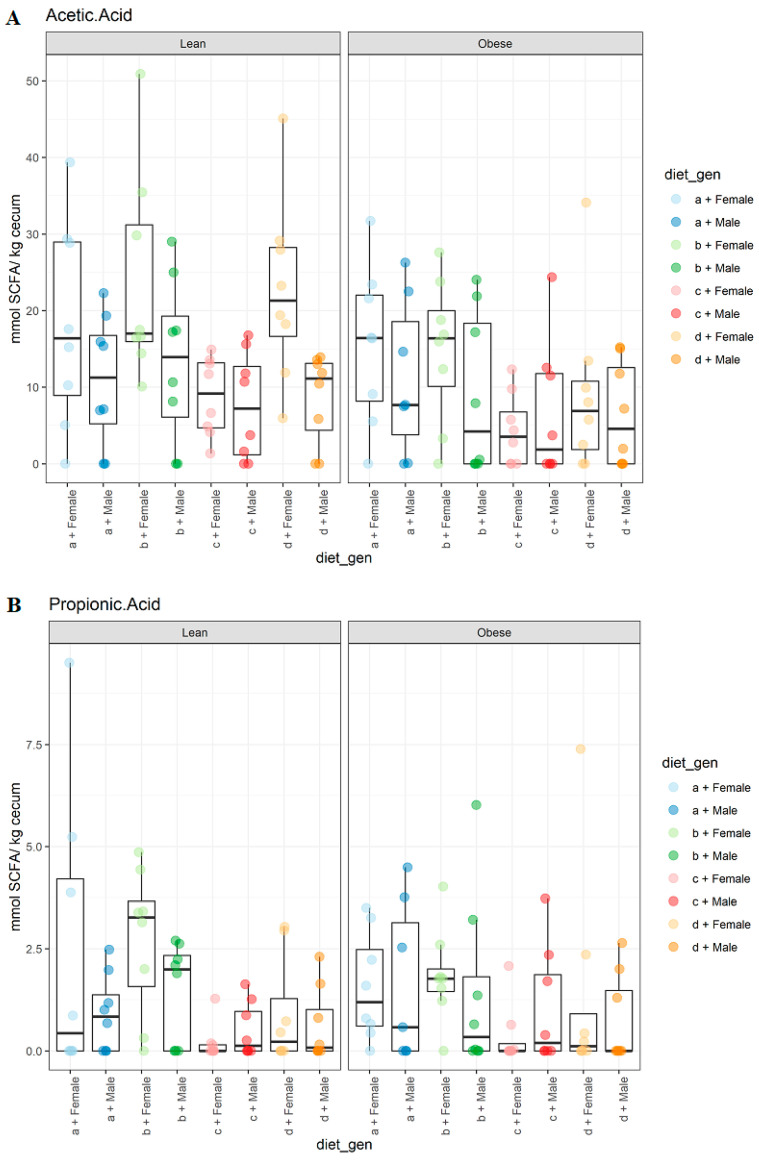
The effect of genotype, sex, and diet on the SCFA content. (**A**), acetic acid levels, genotype (*p* < 0.008), diet (*p* < 0.004), sex (*p* < 0.002); (**B**), propionic acid levels, diet (*p* < 0.004). *n* = 8.

The gut microbiota impacts host physiology by fermenting dietary fiber and producing SCFAs. Thus, we investigated the effect of genotype, sex, and diet on the SCFAs content in the fecal pellets of JCR:LA-*cp* rats. The fecal SCFAs detected by GC-FID were acetate, propionate, isobutyrate, butyrate, isovalerate, valerate, and hexanoate. For acetic acid, there was a significant effect (*p* < 0.008) of genotype as obese animals had lower values compared to their lean counterparts (Figure 5A). Diet (*p* < 0.004) and sex (*p* < 0.002) as factors also had a significant effect on the acetic acid levels. JCR:LA-*cp* rats of both genotypes on the HFHS diet had lower values compared to the control and CFlax diets. Further, male animals had lower levels compared to female animals on all diet groups. A similar effect of diet (*p* < 0.004) was observed for propionic acid as rats of both genotypes on the HFHS diet had lower values compared to the animals on control and CFlax diets (Figure 5B). However, no significant differences were observed for genotype and sex.

**Figure 6:**
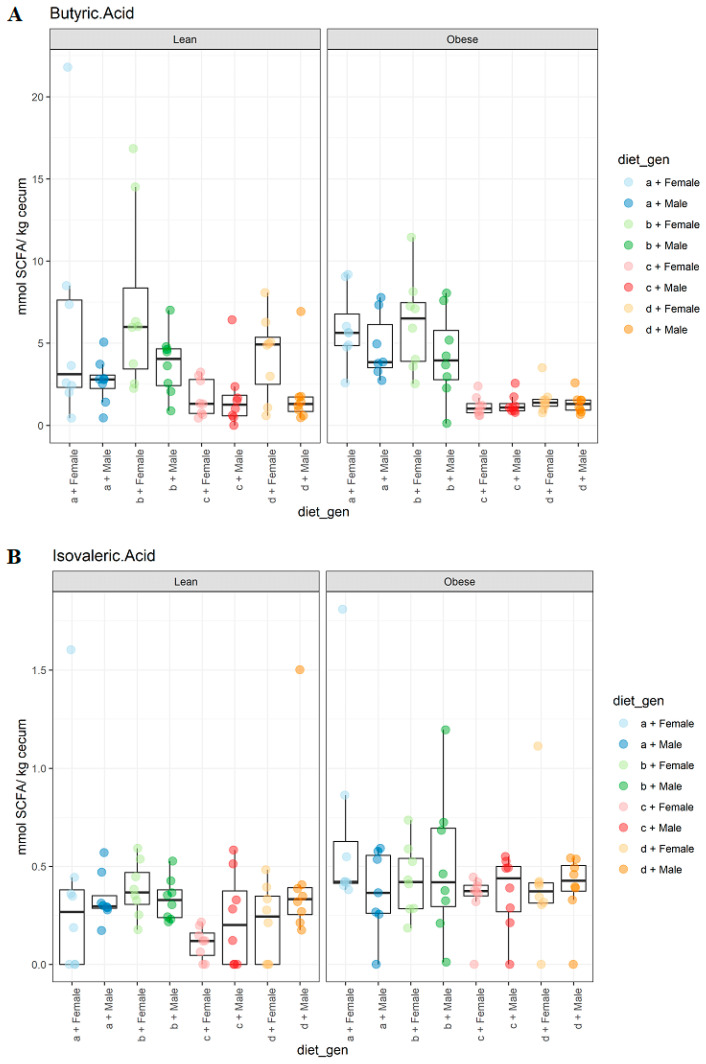
The effect of genotype, sex, and diet on the SCFA content. (**A**), butyric acid levels, sex (*p* < 0.002), diet (*p* < 0.000); (**B**), isovaleric acid levels, genotype (*p* < 0.009). *n* = 8.

**Figure 7:**
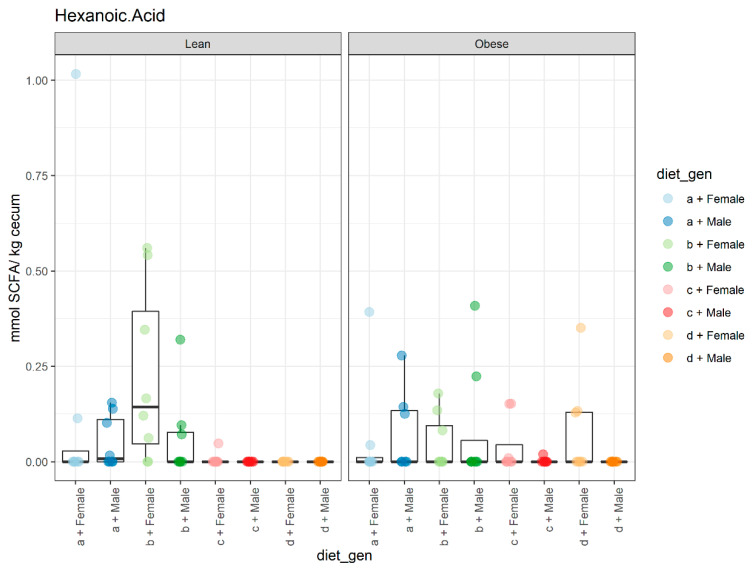
The effect of genotype, sex, and diet on the hexanoic acid content. Diet (*p* < 0.013). *n* = 8.

A significant effect of sex (*p* < 0.002) was observed for the butyric acid values as females had higher values than males and animals on the HFHS, and there was also a significant effect from diet (*p* < 0.000), as HFlax diets had lower values compared to the control and CFlax diets (Figure 6A). No changes were reported for genotype. Although no changes were noted for the effect of diet and sex on the isovaleric acid values, genotype had a significant effect (*p* < 0.009), as obese animals had higher values compared to their lean counterparts (Figure 6B). Diet had a significant effect (*p* < 0.013) on hexanoic acid levels as animals on the HFHS diet had lower values compared to the animals on the CFlax diet (Figure 7). No effect of genotype, diet, or sex was observed on isobutyric acid and valeric acid levels (Figure S11A,B).

## 4. Discussion

In the present study, the impact of diet, sex, and genotype on the gut microbiota of JCR:LA-*cp* rats was examined. The results demonstrate that sex alters the microbial composition of the gut and a HFHS diet induces significant differences in the gut microbiota diversity and SCFA profile of both male and female animals. Flaxseed supplementation improved the taxonomic abundance in both sexes of obese animals.

The richness and diversity of the gut microbiota is a factor in determining health and obesity. Greater bacterial richness and diversity is typically associated with better health [4]. The present findings demonstrate that male and female JCR:LA-*cp* rats have a different degree of alpha-diversity (evenness and richness) of gut microbiota. The overall β-diversity of bacteria types in the gut differed between sex as well. The bacterial population was mostly from the phyla Firmicutes, Bacteroidetes and Verrucomicrobia, with Actinobacteria and Proteobacteria also present in lower proportions. Although obese male rats had similar degree of alpha-diversity as lean males, obese females had substantially lower alpha-diversity than lean females for all diets. This indicates that genetic obesity alters the composition of the gut microbiota in female JCR:LA-*cp* rats, as obese females have less bacterial richness and diversity than the lean females.

Diet greatly affected the diversity of gut microbiota as the HFHS diet showed lowered diversity than control diets. Diet can modify the gut microbiota of humans [17] and rodents [39] very rapidly. Here, the rats were fed HFHS for a longer period of time (12 weeks); thus, the alpha-diversity of the gut microbiota of rats fed the HFHS diet was dramatically altered and the β-diversity plot showed a huge difference between HFHS and control diets. An HFHS diet is often associated with a decrease in bacteria from the phylum Bacteroidetes and an increase in bacteria from the phylum Firmicutes [16]. In our study, there were two species belonging to the family Muribaculaceae (previously known as S24-7) from the phylum Bacteroidetes that were significantly lower in animals fed the HFHS diet. Many bacteria from the phylum Firmicutes increased in abundance in rats fed HFHS including *Ruminococcus gnavus*, which is linked to Crohn’s disease [40] and *Clostridium cocleatum*, which is positively correlated with LPS, common in patients with chronic liver disease [41]. These animals demonstrated some evidence of liver disease (unpublished observations). Conversely, several genera from the phylum Firmicutes were reduced in abundance due to the HFHS diet. At the genus level, Oscillospira showed a reduction in abundance in rats fed the HFHS diet regardless of genotype. Oscillospira may be associated with a steady and healthy gut microbiota [42]. A decrease in a Lactobacillus species was also observed, a SCFA producing genus that is generally considered to be part of a healthy microflora [43]. Overall, the HFHS diet reduced the abundance of several bacterial genera deemed beneficial and increased the quantity of bacteria associated with certain diseases.

The HFHS diet-induced dysbiosis of the gut microbiota was not completely ameliorated by the addition of flaxseed to the diet, yet there were several significant improvements in the abundance of certain bacterial species of interest. The HFHS diet caused a large increase of a *Blautia* species compared to the control diets in both males and females regardless of genotype. However, when flaxseed was added into the diet, the amount of *Blautia* returned to control levels. This is an interesting finding, as the genus Blautia has been positively associated with visceral fat (VF) accumulation in humans [44]. Possibly dietary intake of flaxseed could lessen the quantity of Blautia in the intestinal track, which may result in a decrease of VF. There was also an increase in abundance of the bacteria *Eubacterium dolichum* when rats were fed the HFHS diet. *E. dolichum* has similarly been associated with VF via an unhealthy diet [45]. The addition of flax lowered the abundance of *E. dolichum* for both males and females, but only in the obese genotype. VF accumulation is a major factor in metabolic and cardiovascular diseases [46]. It is encouraging that flax supplementation can reduce the abundance of bacteria related to VF. The genus Allobaculum has previously been shown to be less abundant on a high fat diet compared to a low fat diet [47]. The present results demonstrated that the abundance of Allobaculum was slightly lower in the HFHS diets, but it was dramatically increased with the CFlax diet. Allobaculum, which has been positively correlated with butyrate production [48], may be beneficial for host physiology and is associated with energy homeostasis [49]. The abundance of a *Dorea* species was also increased in lean and obese rats fed a HFHS diet supplemented with flax. Although found in a normal healthy gut microbiota, an abundance of Dorea and Blautia are found in alcohol-dependent subjects with high intestinal permeability [50]. Even though the abundance of Dorea was increased with flax, it was only increased with the HFHS diet. This suggests that some of the potential beneficial aspects of flax consumption may be diminished when taken in conjunction with an unhealthy diet. This demonstrates the complex challenge researchers face when trying to determine which prebiotics regulate the quantities of which bacteria and what abundances of these bacteria are important for human health. We conclude that flax has an effect on the JCR:LA-*cp* rat gut microbiota at the genus level, decreasing the numbers of potentially unhealthy bacteria, and potentially improving the health of the gut.

The association between obesity, diet, and SCFAs produced by gut microbiota is not yet fully understood. Intestinal bacteria are known to produced SCFAs including acetate, propionate, and butyrate as crucial end-products by fermenting dietary fibers [51]. Up to 200 kcal/day of human energy can be attributed to these SCFAs [52]. SCFAs accumulate in adipocytes and contribute to lipogenesis [53]. The SCFAs exert their biological effects by interacting with G-protein coupled receptors (GPR41 or GPR43, which are also known as free fatty acid receptor 3 and 2, respectively) [54]. Obesity and diets rich in high carbohydrate attenuate the binding of SCFAs to GPRs, consequently leading to impaired intestinal energy harvest and hepatic lipogenesis [55,56,57]. Alterations in SCFAs concentration may be related to gut dysbiosis, gut permeability, obesity, and other cardiovascular risk factors [58]. However, changes in SCFA amounts are typically related to alterations in the gut bacterial community due to diet [59].

Our data revealed altered levels of main SCFAs such as acetate, propionate, and butyrate in the JCR:LA-*cp* rat strain. Acetate is the major SCFA found in the gut. Pathways for acetate production pathways are broadly distributed between bacteria [60]. Murphy et al. investigated the relationship between obesity, diet, and time on the gut microbiota. The fecal acetate levels were shown to be higher in *ob/ob* (leptin-deficient) and high-fat-fed mice at age 7 and 11 weeks compared to their lean counterparts. Conversely, the levels dropped in 15-week old animals [61]. This indicates that the acetate levels decrease progressively over time. Our data aligns with this previous observation, as animals had lower fecal acetate levels when analyzed at 24 weeks of age. These alterations could be due either to gut dysbiosis, or an increase in the acetate uptake or absorption in response to genetic obesity or a high-fat diet consumption [61]. Acetate activates the tricarboxylic acid cycle and changes the expression profile of hypothalamic neuropeptides that suppress appetite [62]. The decreased acetate levels thus support the hyperphagic behavior of obese animals in this study. Propionate administration to obese patients reduced excess adiposity and overall weight gain by enhancing the secretion of glucagon-like peptide-1 and gut hormone peptide YY [63]. The decreased levels of propionate in the HFHS group observed in the present study may have impacted the body weight. However, the association between the increased body weight and the lowered fecal propionate levels in the JCR:LA-*cp* rats that received a HFHS diet need a valid assessment in further studies. Butyrate is generally considered to be beneficial to human health including maintenance of the colonic epithelium [60]. The butyric acid levels in an animal study has been reported to prevent the translocation of lipopolysaccharide (LPS), which is a potent inflammatory molecule originating in the cell membrane of gram-negative bacteria. Through its adverse inflammatory effect, LPS can cause metabolic endotoxemia, insulin resistance, and obesity [64]. The decreased fecal levels of butyrate observed in the HFHS diet groups in our study could thus explain the metabolic abnormalities observed in these animals.

Bacteroidetes usually contribute to acetate and propionate production, whereas Firmicutes mainly produce butyrate [65]. The abundance of Muribaculaceae from the phylum Bacteroidetes has been strongly associated with propionate levels [66]. Members of Ruminococcus, from the phylum Firmicutes, have been related to increased butyrate concentrations [66]. Consistent with these earlier observations, our study also found corresponding changes in SCFA with altered abundances of Bacteroidetes and Firmicutes.

It was unclear previously if SCFAs contribute to obesity or reflect the gut dysbiosis due to obesity. Our finding in the JCR:LA-*cp* rat strain of genetic obesity has unraveled this novel and interesting association. There were no differences in the fecal levels of propionate and butyrate among obese and lean genotypes. However, both genotypes showed altered levels of the main SCFAs such as acetate, propionate, and butyrate when administered an HFHS diet compared to the Con diet. Unless maintained on an HFHS diet, the obese genotype had similar levels of SCFAs as their lean counterparts. Therefore, established genetic obesity does not impact SCFAs. Instead, a western diet known to contribute to excess adiposity alters SCFAs which may subsequently affect energy homeostasis and cause weight gain. In summary, the dysbiosis of the gut microbiota caused by an HFHS diet is reflected in the SCFA profile. This study demonstrates that the gut microbiota is modified due to sex and genotype. We also show that an unhealthy diet leads to a dysbiosis of gut microbiota and a reduction of SCFA, demonstrating that the microbial composition of the gut is very dynamic. Supplementing a healthy diet with prebiotics, such as flaxseed, can establish and enhance a healthy microbial production in the human gut, which in turn can lead to production of healthy SCFAs.

## Data Availability

Data is contained within the article and/or Supplementary Material. The data presented in this study are available in this article or the Supplementary Material here or on-line.

## Supplementary Materials

The following are available online at https://www.mdpi.com/article/10.3390/microorganisms9051037/s1, Figure S1: Analytical flowchart describing data processing and analysis, Figure S2: The box-and-whisker plot illustrates the total number of quality filtered per sample, Figure S3: The plot illustrates the mean and standard error of the relative abundances of the 5 most abundant genus-level taxa within the 4 most abundant Phyla, Figure S4: Taxonomic composition at the class level, Figure S5: Taxonomic composition at the order level, Figure S6: Taxonomic composition at the family level, Figure S7: Taxonomic composition at the species level, Figure S8: Differential abundance of Subdolingranulum variabile as a function of dietary interventions in lean and obese, male and female, JCR:LA cp rats fed different diets, Figure S9: Differential abundance of a SMB53 species as a function of dietary interventions in lean and obese, male and female, JCR:LA cp rats fed different diets, Figure S10: Differential abundance of an unclassified species from the family Lachnospiraceae as a function of dietary interventions in lean and obese, male and female, JCR:LA cp rats fed different diets, Figure S11: The effect of genotype, sex, and diet on the SCFA content, Table S1: The following table summarizes the total number of quality filtered per sample.
