# Supplementary material for: The Influence of Diet and Sex on the Gut Microbiota of Lean and Obese JCR:LA-cp Rats
Source: Microorganisms. 2021 May 12;9(5):1037. doi: 10.3390/microorganisms9051037 (PMC8151891; doi:10.3390/microorganisms9051037)
Supplement: Supplementary file 1 [file microorganisms-09-01037-s001.zip › microorganisms-1126095-supplementary.pdf]

Supplementary Materials

Table S1. The following table summarizes the total number of quality filtered per sample.

MBI.Sample.ID	Your.Sample.ID	Gender	Diet	Genotype	Reads
S009I-0001	18RAT0332	Male	a	Lean	46996
S009I-0002	18RAT0333	Male	a	Lean	46970
S009I-0003	18RAT0334	Male	b	Lean	47331
S009I-0004	18RAT0335	Male	b	Lean	64317
S009I-0005	18RAT0336	Male	c	Lean	121214
S009I-0006	18RAT0342	Male	c	Lean	45156
S009I-0007	18RAT0343	Male	d	Lean	40887
S009I-0008	18RAT0344	Male	d	Lean	53398
S009I-0009	18RAT0374	Female	a	Lean	36497
S009I-0010	18RAT0375	Female	a	Lean	43897
S009I-0011	18RAT0376	Female	b	Lean	64576
S009I-0012	18RAT0377	Female	b	Lean	46379
S009I-0013	18RAT0378	Female	c	Lean	38456
S009I-0014	18RAT0340	Female	c	Lean	111155
S009I-0015	18RAT0379	Female	d	Lean	295
S009I-0016	18RAT0380	Female	d	Lean	476
S009I-0017	18RAT0368	Female	a	Obese	72357
S009I-0018	18RAT0369	Female	a	Obese	39223
S009I-0019	18RAT0370	Female	b	Obese	71309
S009I-0020	18RAT0371	Female	b	Obese	58457
S009I-0021	18RAT0349	Female	c	Obese	62259
S009I-0022	18RAT0372	Female	c	Obese	119694

S009I-0023	18RAT0338	Female	d	Obese	43180
S009I-0024	18RAT0373	Female	d	Obese	61395
S009I-0025	18RAT0361	Male	a	Obese	34628
S009I-0026	18RAT0362	Male	b	Obese	44626
S009I-0027	18RAT0363	Male	b	Obese	49870
S009I-0028	18RAT0364	Male	c	Obese	48761
S009I-0029	18RAT0365	Male	c	Obese	74299
S009I-0030	18RAT0366	Male	d	Obese	69412
S009I-0031	18RAT0367	Male	d	Obese	45537
S009I-0032	18RAT0457	Male	a	Obese	49357
S009I-0033	18RAT0458	Male	a	Obese	58006
S009I-0034	18RAT0459	Male	b	Obese	49958
S009I-0035	18RAT0460	Male	b	Obese	62540
S009I-0036	18RAT0461	Male	c	Obese	57154
S009I-0037	18RAT0462	Male	c	Obese	36024
S009I-0038	18RAT0463	Male	d	Obese	44141
S009I-0039	18RAT0464	Male	d	Obese	44086
S009I-0040	18RAT0473	Female	a	Obese	38391
S009I-0041	18RAT0474	Female	a	Obese	45537
S009I-0042	18RAT0475	Female	b	Obese	76892
S009I-0043	18RAT0476	Female	b	Obese	40121
S009I-0044	18RAT0477	Female	c	Obese	47726
S009I-0045	18RAT0478	Female	c	Obese	55052
S009I-0046	18RAT0479	Female	d	Obese	39949
S009I-0047	18RAT0480	Female	d	Obese	48613
S009I-0048	18RAT0465	Male	a	Lean	34428
S009I-0049	18RAT0466	Male	a	Lean	46229

S009I-0050	18RAT0467	Male	b	Lean	67566
S009I-0051	18RAT0468	Male	b	Lean	53819
S009I-0052	18RAT0469	Male	c	Lean	51454
S009I-0053	18RAT0470	Male	c	Lean	34605
S009I-0054	18RAT0471	Male	d	Lean	39420
S009I-0055	18RAT0472	Male	d	Lean	35997
S009I-0056	18RAT0481	Female	a	Lean	38414
S009I-0057	18RAT0482	Female	a	Lean	42739
S009I-0058	18RAT0483	Female	b	Lean	53222
S009I-0059	18RAT0484	Female	b	Lean	32490
S009I-0060	18RAT0485	Female	c	Lean	25061
S009I-0061	18RAT0486	Female	c	Lean	59087
S009I-0062	18RAT0487	Female	d	Lean	24349
S009I-0063	18RAT0488	Female	d	Lean	63114
S009I-0064	18RAT0577	Male	a	Lean	33327
S009I-0065	18RAT0578	Male	a	Lean	33901
S009I-0066	18RAT0579	Male	b	Lean	47605
S009I-0067	18RAT0580	Male	b	Lean	32401
S009I-0068	18RAT0581	Male	c	Lean	39235
S009I-0069	18RAT0582	Male	c	Lean	34413
S009I-0070	18RAT0583	Male	d	Lean	46679
S009I-0071	18RAT0584	Male	d	Lean	51561
S009I-0072	18RAT0585	Female	a	Lean	41575
S009I-0073	18RAT0586	Female	a	Lean	57420
S009I-0074	18RAT0587	Female	b	Lean	37787
S009I-0075	18RAT0588	Female	b	Lean	41538
S009I-0076	18RAT0589	Female	c	Lean	37273

S009I-0077	18RAT0590	Female	c	Lean	45098
S009I-0078	18RAT0591	Female	d	Lean	25800
S009I-0079	18RAT0592	Female	d	Lean	27801
S009I-0080	18RAT0593	Female	a	Obese	37291
S009I-0081	18RAT0594	Female	a	Obese	54322
S009I-0082	18RAT0595	Female	b	Obese	36322
S009I-0083	18RAT0596	Female	b	Obese	48821
S009I-0084	18RAT0597	Female	c	Obese	33257
S009I-0085	18RAT0598	Female	c	Obese	48447
S009I-0086	18RAT0599	Female	d	Obese	35870
S009I-0087	18RAT0600	Female	d	Obese	43832
S009I-0088	18RAT0601	Male	a	Obese	27070
S009I-0089	18RAT0602	Male	a	Obese	51474
S009I-0090	18RAT0603	Male	b	Obese	30849
S009I-0091	18RAT0604	Male	b	Obese	28819
S009I-0092	18RAT0605	Male	c	Obese	38415
S009I-0093	18RAT0606	Male	c	Obese	38112
S009I-0094	18RAT0607	Male	d	Obese	23994
S009I-0095	18RAT0608	Male	d	Obese	40231
S009I-0096	18RAT0658	Male	a	Lean	45504
S009I-0097	18RAT0659	Male	a	Lean	56335
S009I-0098	18RAT0660	Male	b	Lean	33367
S009I-0099	18RAT0661	Male	b	Lean	35225
S009I-0100	18RAT0662	Male	c	Lean	31691
S009I-0101	18RAT0663	Male	c	Lean	38992
S009I-0102	18RAT0664	Male	d	Lean	30233
S009I-0103	18RAT0665	Male	d	Lean	25554

S009I-0104	18RAT0666	Female	a	Lean	25446
S009I-0105	18RAT0667	Female	a	Lean	29319
S009I-0106	18RAT0668	Female	b	Lean	23419
S009I-0107	18RAT0669	Female	b	Lean	36539
S009I-0108	18RAT0670	Female	c	Lean	38714
S009I-0109	18RAT0671	Female	c	Lean	42667
S009I-0110	18RAT0672	Female	d	Lean	50083
S009I-0111	18RAT0673	Female	d	Lean	63589
S009I-0112	18RAT0682	Male	a	Obese	29011
S009I-0113	18RAT0683	Male	a	Obese	52564
S009I-0114	18RAT0684	Male	b	Obese	36651
S009I-0115	18RAT0685	Male	b	Obese	31486
S009I-0116	18RAT0686	Male	c	Obese	22412
S009I-0117	18RAT0687	Male	c	Obese	30902
S009I-0118	18RAT0688	Male	d	Obese	22971
S009I-0119	18RAT0689	Male	d	Obese	32108
S009I-0120	18RAT0674	Female	a	Obese	23046
S009I-0121	18RAT0675	Female	a	Obese	49098
S009I-0122	18RAT0676	Female	b	Obese	29370
S009I-0123	18RAT0677	Female	b	Obese	42692
S009I-0124	18RAT0678	Female	c	Obese	37471
S009I-0125	18RAT0679	Female	c	Obese	40234
S009I-0126	18RAT0680	Female	d	Obese	53150
S009I-0127	18RAT0681	Female	d	Obese	62214

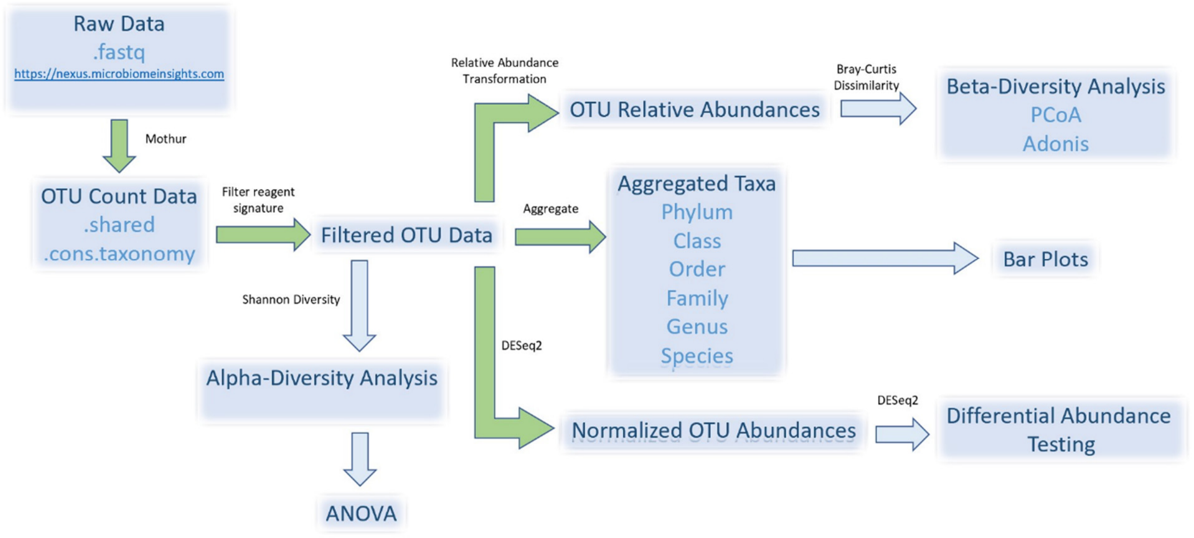

Figure S1. Analytical flowchart describing data processing and analysis.

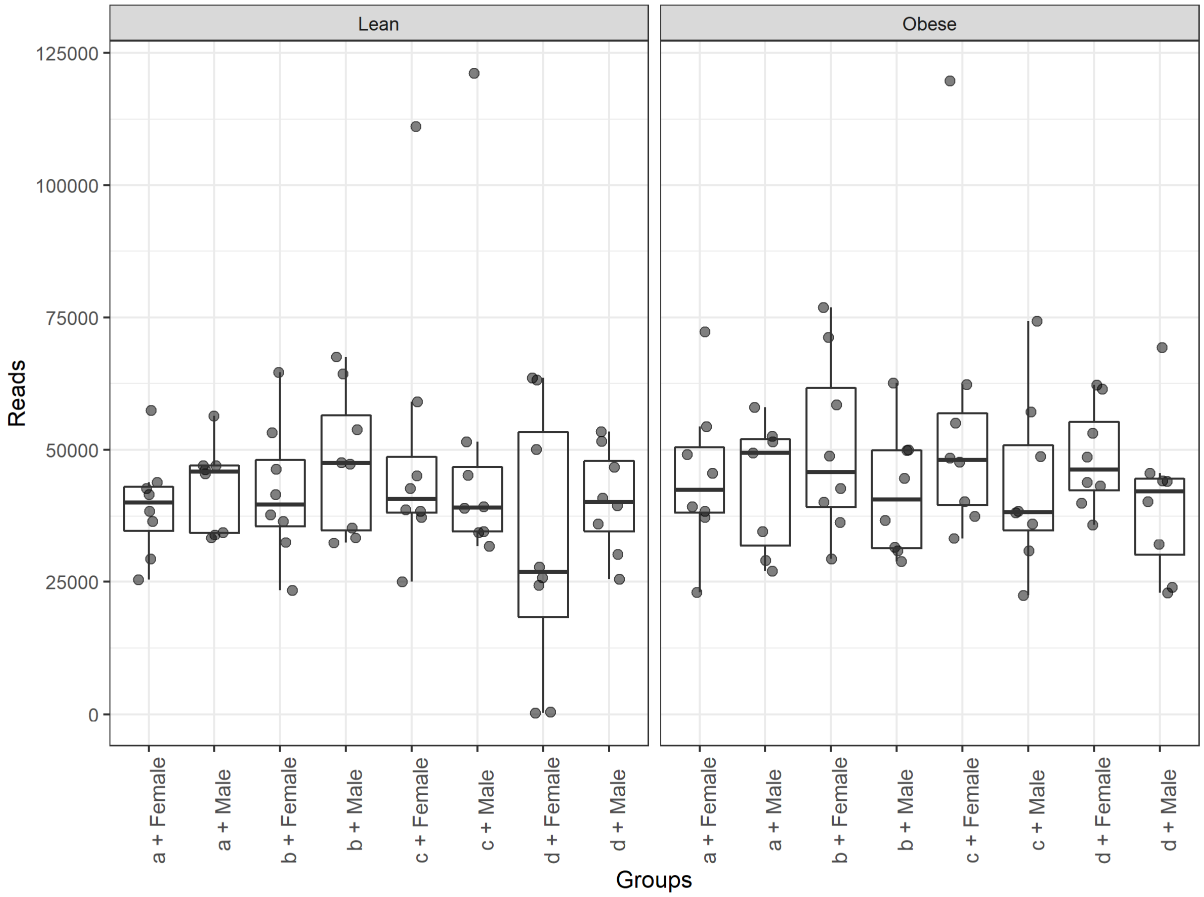

Figure S2. The box-and-whisker plot illustrates the total number of quality filtered per sample. These reads reflect the total number of high-quality sequences that align with 16Sv4,

clustered into OTUs and were assigned taxonomic classification. Any ambiguous or low-quality data were discarded from the subsequent analyses.

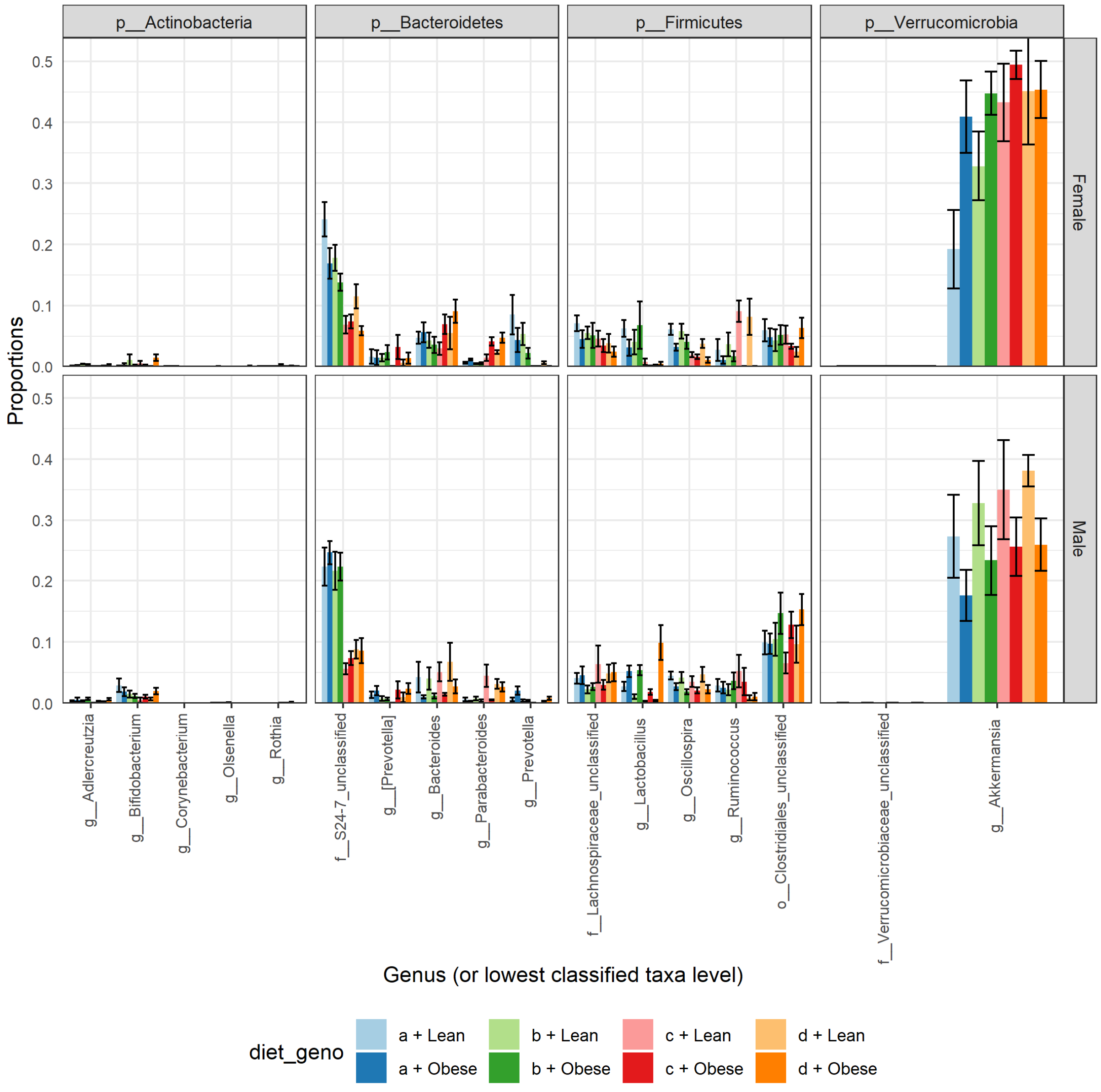

Figure S3. The plot illustrates the mean and standard error of the relative abundances of the 5 most abundant genus-level taxa within the 4 most abundant Phyla. The Genus-level plots are grouped according to Phylum along the x-axis. The groupings along the y-axis represent the sex column of metadata. The bar plot colors represent diet/genotype.

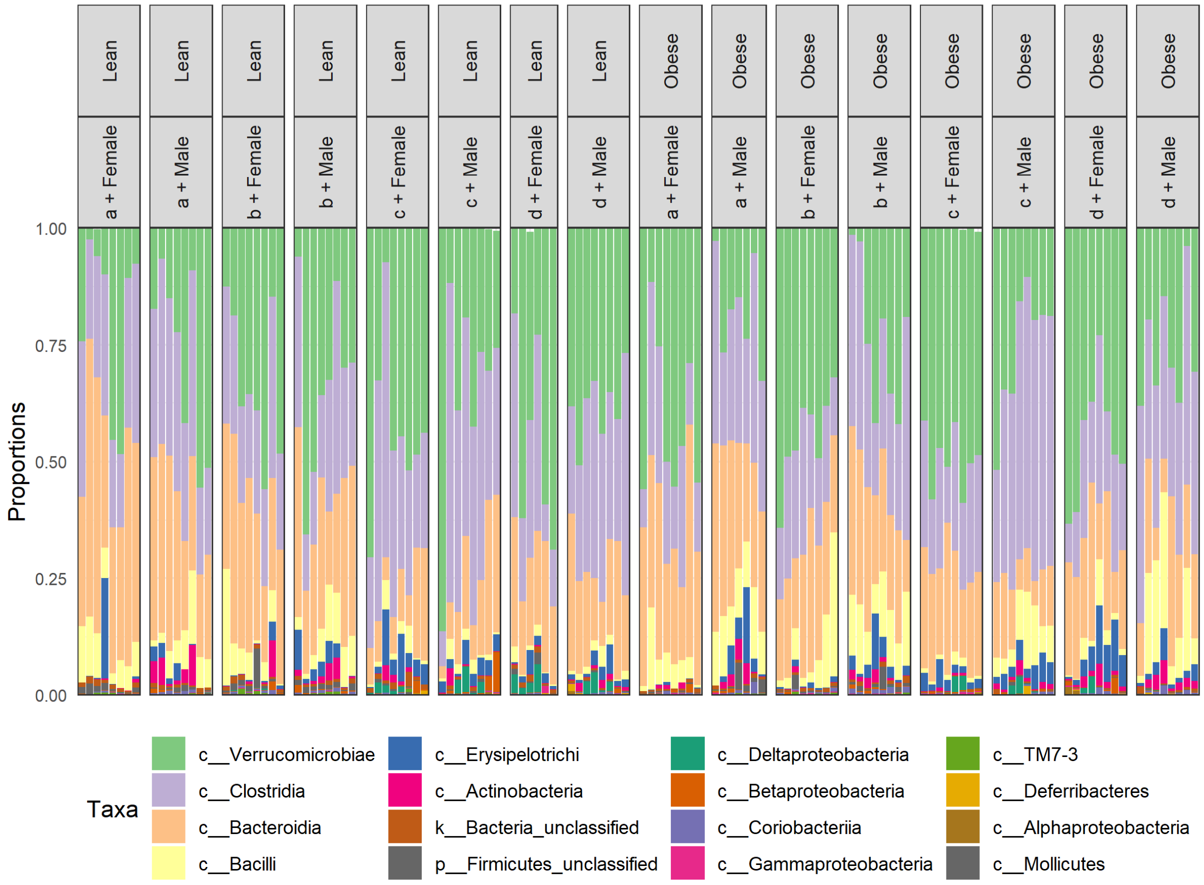

Figure S4. Taxonomic composition at the class level. The aggregated taxa were visualized at each taxonomic rank using the taxonomic bar plots. The unfilled portion of the bar plots represent lower-abundance taxa.

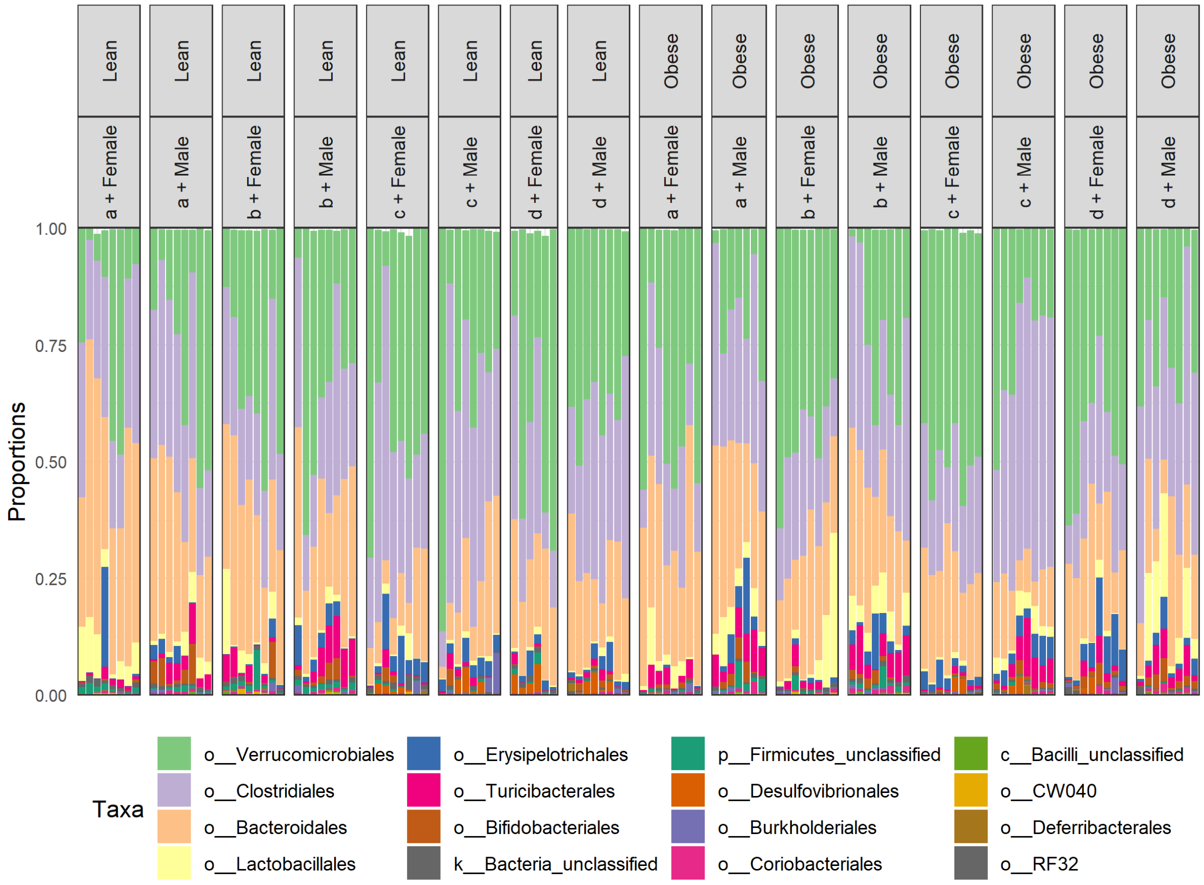

Figure S5. Taxonomic composition at the order level. The aggregated taxa were visualized at each taxonomic rank using the taxonomic bar plots. The unfilled portion of the bar plots represent lower-abundance taxa.

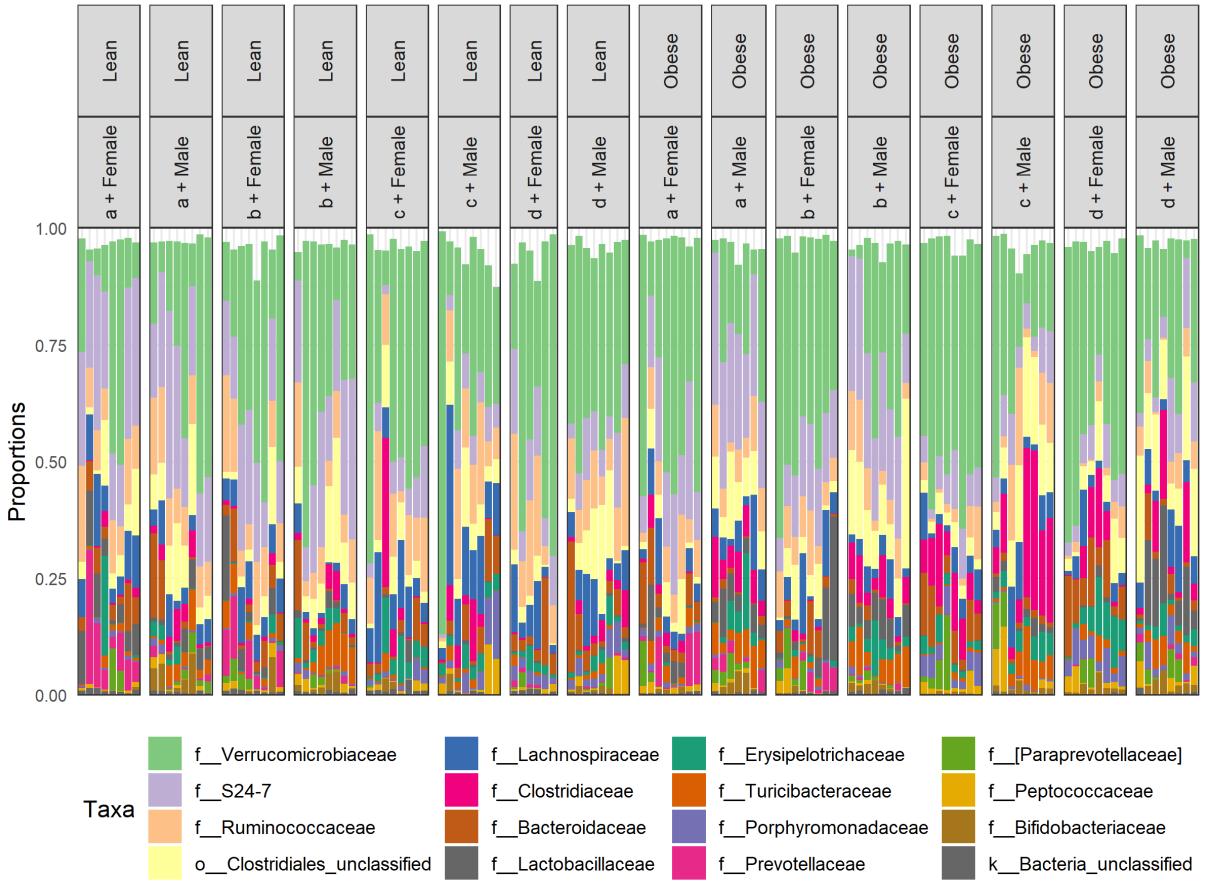

Figure S6. Taxonomic composition at the family level. The aggregated taxa were visualized at each taxonomic rank using the taxonomic bar plots. The unfilled portion of the bar plots represent lower-abundance taxa.

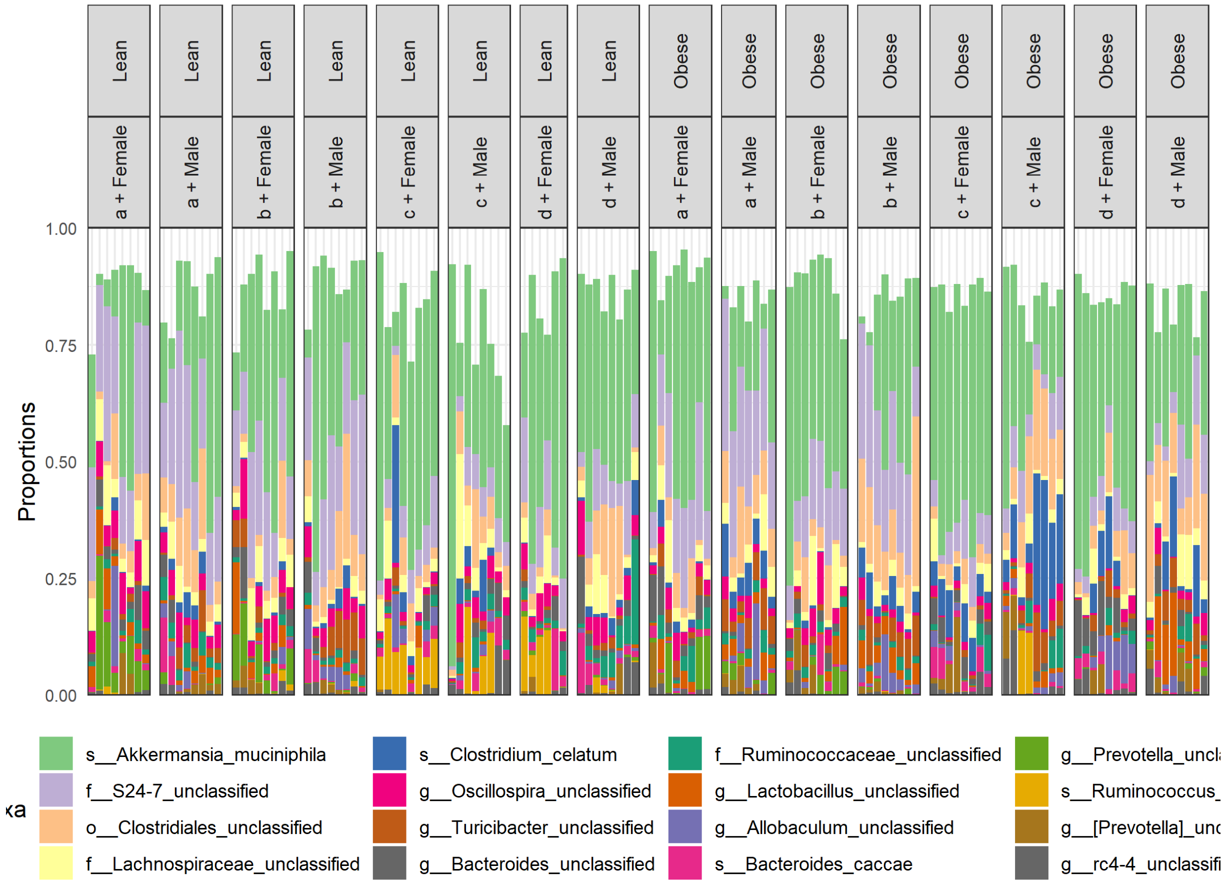

Figure S7. Taxonomic composition at the species level. The aggregated taxa were visualized at each taxonomic rank using the taxonomic bar plots. The unfilled portion of the bar plots represent lower-abundance taxa.

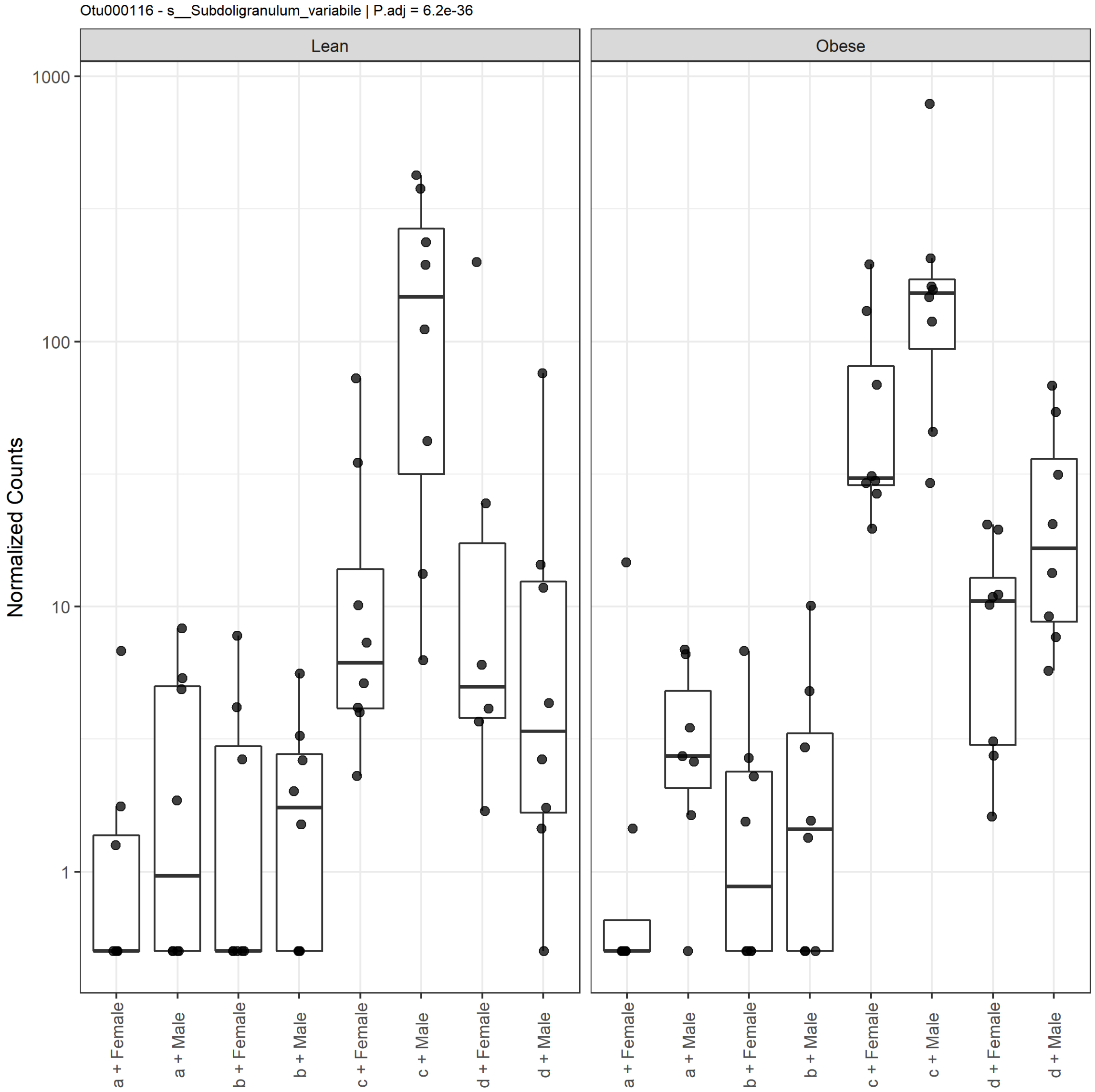

Figure S8. Differential abundance of *Subdolingranulum variabile* as a function of dietary interventions in lean and obese, male and female, JCR:LA cp rats fed a) control diet, b) control + flax, c) high fat, high sucrose or d) high fat, high sucrose + flax. N=8.

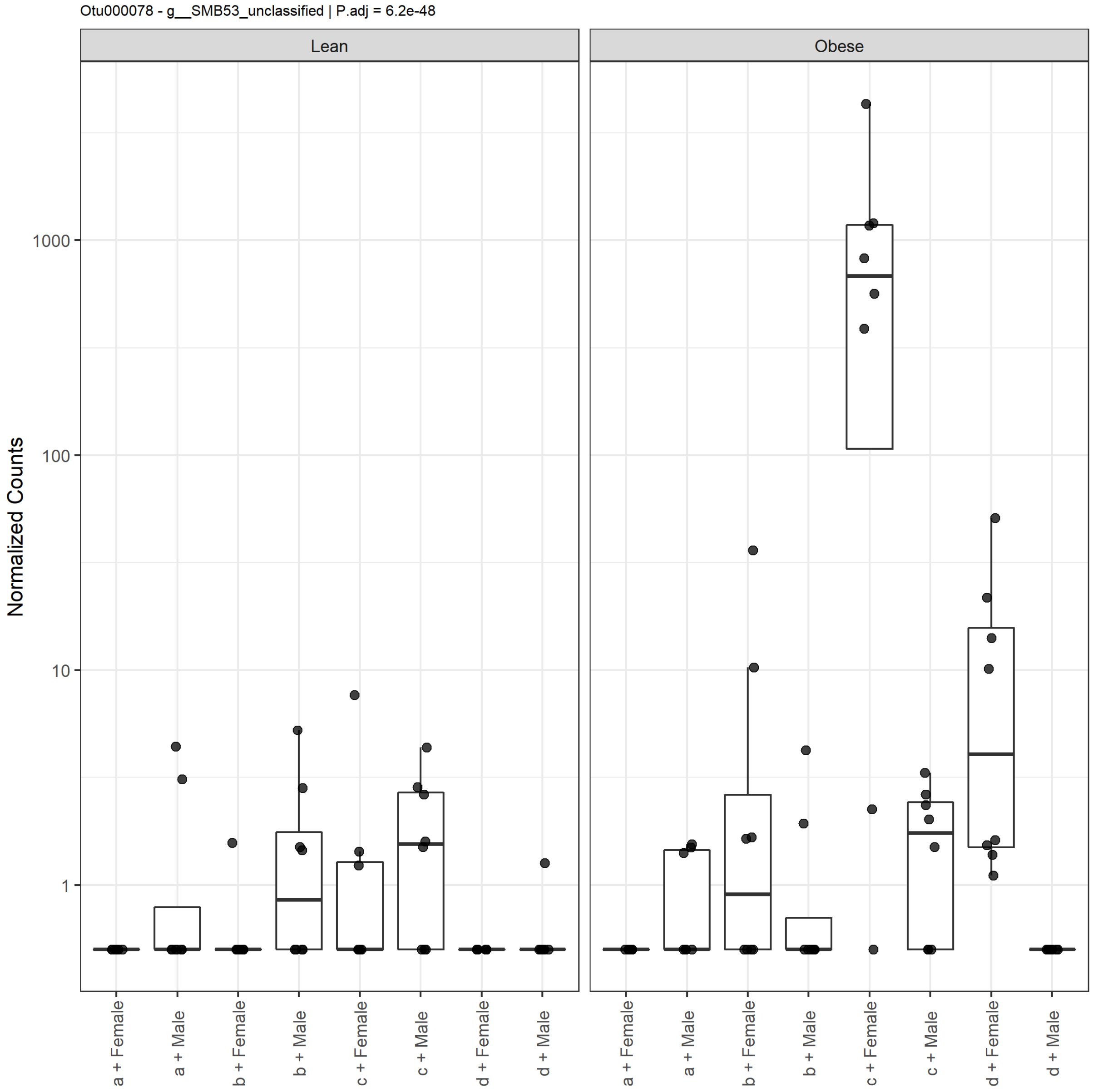

Figure S9. Differential abundance of a *SMB53* species as a function of dietary interventions in lean and obese, male and female, JCR:LA cp rats fed a) control diet, b) control + flax, c) high fat, high sucrose or d) high fat, high sucrose + flax. N=8.

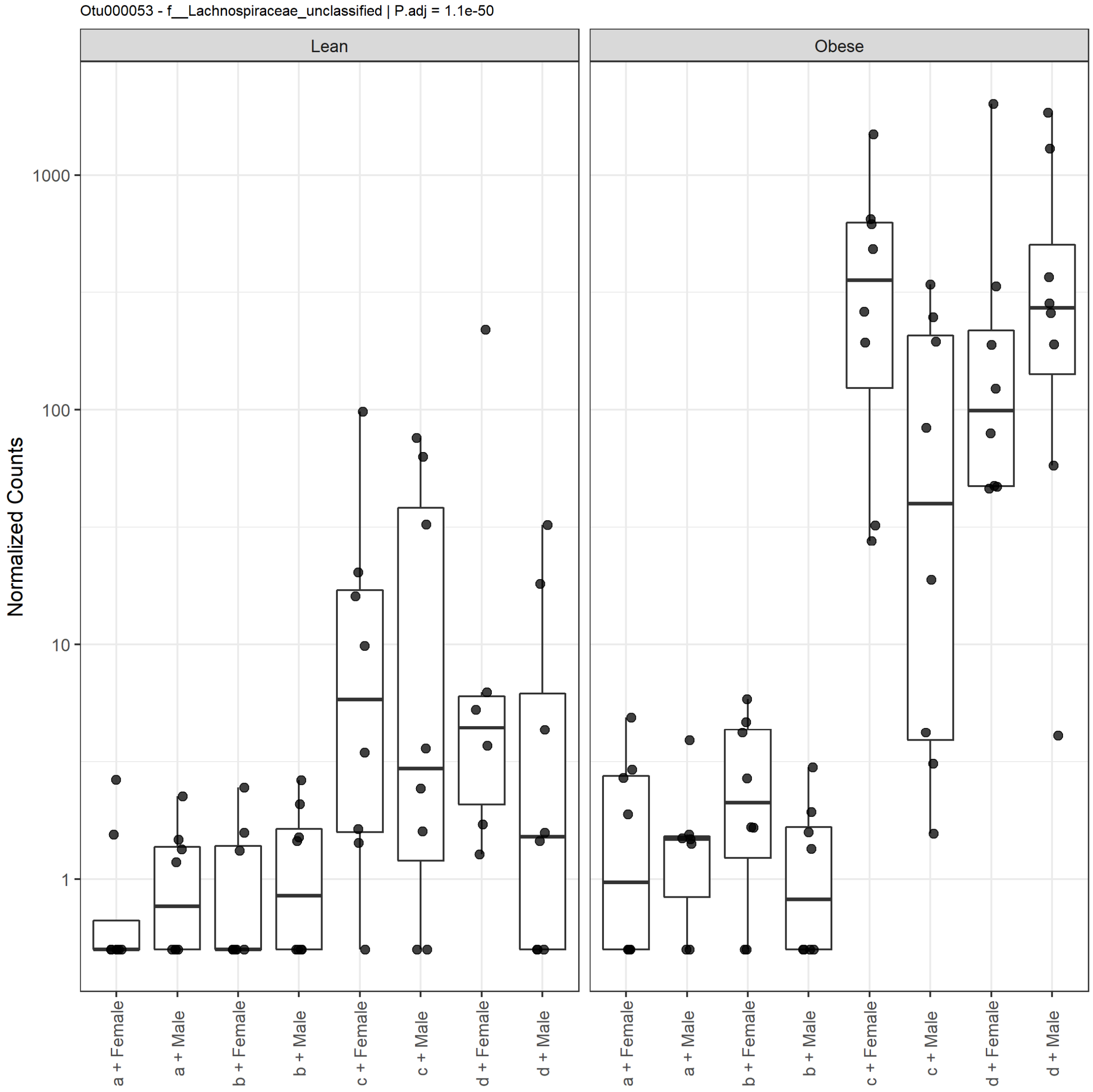

Figure S10. Differential abundance of an unclassified species from the family *Lachnospiraceae* as a function of dietary interventions in lean and obese, male and female, JCR:LA cp rats fed a) control diet, b) control + flax, c) high fat, high sucrose or d) high fat, high sucrose + flax. N=8.

A.

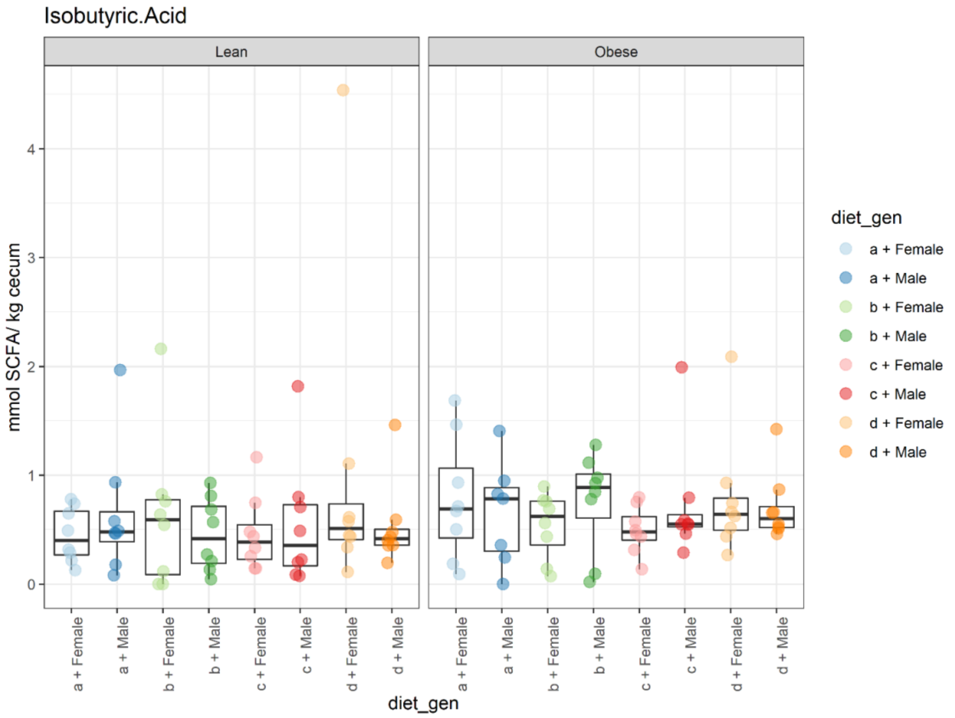

B.

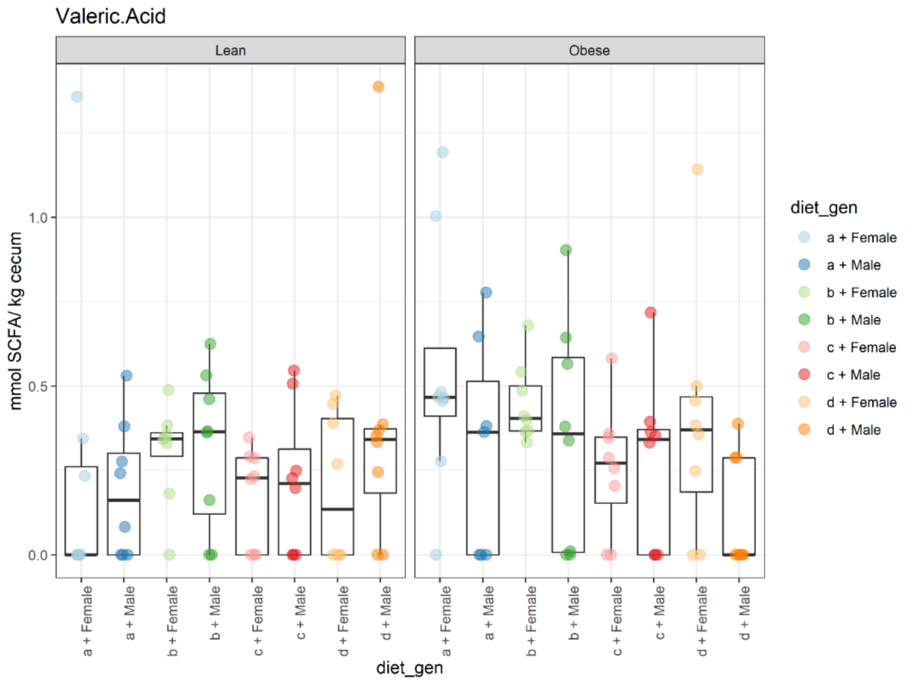

Figure S11. The effect of genotype, sex, and diet on the SCFA content. **A**, isobutyric acid levels; **B**, valeric acid levels.
